# The Vacuolar Molybdate Transporter OsMOT1;2 Controls Molybdenum Remobilization in Rice

**DOI:** 10.3389/fpls.2022.863816

**Published:** 2022-03-09

**Authors:** Dawei Hu, Mengzhen Li, Fang-Jie Zhao, Xin-Yuan Huang

**Affiliations:** State Key Laboratory of Crop Genetics and Germplasm Enhancement, College of Resources and Environmental Sciences, Nanjing Agricultural University, Nanjing, China

**Keywords:** rice, molybdenum, remobilization, vacuole, exporter

## Abstract

Molybdenum (Mo) is an essential micronutrient for almost all living organisms. The Mo uptake process in plants has been well investigated. However, the mechanisms controlling Mo translocation and remobilization among different plant tissues are largely unknown, especially the allocation of Mo to rice grains that are the major dietary source of Mo for humans. In this study, we characterized the functions of a molybdate transporter, OsMOT1;2, in the interorgan allocation of Mo in rice. Heterologous expression in yeast established the molybdate transport activity of OsMOT1;2. *OsMOT1;2* was highly expressed in the blades of the flag leaf and the second leaf during the grain filling stage. Subcellular localization revealed that OsMOT1;2 localizes to the tonoplast. Knockout of *OsMOT1;2* led to more Mo accumulation in roots and less Mo translocation to shoots at the seedling stage and to grains at the maturity stage. The remobilization of Mo from older leaves to young leaves under molybdate-depleted condition was also decreased in the *osmot1;2* knockout mutant. In contrast, overexpression of *OsMOT1;2* enhanced the translocation of Mo from roots to shoots at the seedling stage. The remobilization of Mo from upper leaves to grains was also enhanced in the overexpression lines during grain filling. Our results suggest that OsMOT1;2 may function as a vacuolar molybdate exporter facilitating the efflux of Mo from the vacuole into the cytoplasm, and thus, it plays an important role in the root-to-shoot translocation of Mo and the remobilization of Mo from leaves to grains.

## Introduction

Molybdenum (Mo) is an essential micronutrient for the growth and development of almost all living organisms. Mo participates in physiological and biochemical metabolic processes by incorporating into tricyclic pterin to form the Mo cofactor (Moco), which forms the active site of Mo-requiring enzymes (molybdoenzymes) and facilitates electron transfer (Schwarz and Mendel, 2006; Bittner, 2014). In humans, four main molybdoenzymes, namely, aldehyde oxidase (AO), xanthine dehydrogenase (XDH), sulfite oxidase (SO), and amidoxime-reducing component (ARC), are essential for maintaining human health by participating in purine metabolism, sulfite detoxification, and other crucial processes (Schwarz and Mendel, 2006). A decrease in molybdoenzyme activity due to Moco biosynthesis defect could cause inheritable progressive neurological damage and even early childhood death (Johnson et al., 1980; Schwarz, 2005). The intake of sufficient Mo in the diet is thus vital for human health. The estimated average requirement for Mo is 17–22 μg/day for children and 45–50 μg/day for adults (Trumbo et al., 2001). Cereals are the major dietary source of Mo. Biofortification to increase Mo in eatable parts of staple foods, such as rice, is one promising approach to provide the minimum amount of dietary Mo for humans. However, the mechanisms underlying the translocation of Mo into rice grain are largely unknown.

Molybdenum is also required for the growth and development of plants. In plants, there are five types of molybdoenzymes, namely, nitrate reductase (NR), SO, XDH, AO, and ARC (Schwarz and Mendel, 2006; Tejada-Jimenez et al., 2013). These enzymes participate in crucial processes, such as nitrate assimilation, hormone biosynthesis, purine metabolism, and sulfite detoxification, and they play a vital role in maintaining plant growth and stress resistance. The activities of these enzymes are influenced by Moco synthesis, which depends directly on the Mo supply level in the cells. Mo limitation impairs plant growth and leads to the typical “whiptail” phenotype with mottled lesions on the leaves, rolling of leaves, and wilting of leaf edges (Arnon and Stout, 1939). Mo deficiency is common in plants grown on acid soils where the bioavailability of Mo is decreased due to the strong adsorption of Mo to reactive iron oxides/hydroxides (Marschner and Rengel, 2012). Currently, the global arable land is facing the risk of Mo deficiency because it is estimated that 70% of the world’s arable land is acidic (Von Uexküll and Mutert, 1995). Therefore, improving Mo nutrition is of importance for plant growth and agricultural productivity.

Plants have evolved sophisticated mechanisms to regulate the uptake, translocation, and remobilization of Mo to maintain optimal Mo levels in different tissues. Plant roots take up Mo mainly in the form of molybdate. Previous studies have demonstrated that there are two types of Mo uptake system in plants, namely, a specific and a non-specific system (Tejada-Jimenez et al., 2013; Huang et al., 2021). Sulfate transporters have been reported to have non-specific Mo transport activities. Heterologous expression of *Stylosanthes hamata* sulfate transporter SHST1 in yeast showed a molybdate transport activity (Fitzpatrick et al., 2008); however, more evidence, especially *in vivo* results, is needed to confirm this non-specific transport activity. MOlybdenum Transporter type 1 (MOT1) and type 2 (MOT2) are two Mo-specific transport systems in plants. The first identified member of MOT1 family is CrMOT1, which was identified from the green alga *Chlamydomonas reinhardtii* (Tejada-Jimenez et al., 2007). CrMOT1 transports Mo in a high-affinity way with a *K*m of 6.7 ± 0.6 nM. Knockdown of *CrMOT1* dramatically decreased Mo transport activity and nitrate reductase activity. Subsequently, a member of the MOT1 family in higher plants, AtMOT1;1, was identified as a high-affinity molybdate transporter in *Arabidopsis thaliana* (Tomatsu et al., 2007; Baxter et al., 2008). Compared with wild type, *atmot1;1* has extremely lower Mo concentrations in shoots and roots and is more sensitive to Mo deficiency. Similarly, OsMOT1;1 plays an important role in controlling Mo accumulation in rice (Yang et al., 2018; Huang et al., 2019; Wang et al., 2020). Knockout of *OsMOT1;1* results in a significant decrease of Mo concentration in various tissues of rice plants at the seedling and harvesting stages (Huang et al., 2019). The homologs of AtMOT1;1 have been identified and characterized in other species. In the model legume *Medicago truncatula*, *MtMOT1.3* is specifically expressed in nodules and plays an important role in symbiotic nitrogen fixation, while *MtMOT1.2* is expressed in root and nodules and is responsible for Mo delivery into the nodules by the vasculature (Tejada-Jimenez et al., 2017; Gil-Diez et al., 2019). In another legume *Lotus japonicus*, *LjMOT1* is essential for Mo uptake from the soil and plant growth, but is not involved in the delivery of Mo into nodules (Gao et al., 2016; Duan et al., 2017). Among the MOT2 family, only CrMOT2 in *C. reinhardtii* has so far been confirmed to possess the Mo transport activity (Tejada-Jimenez et al., 2011).

Although the process of uptake of Mo has been well characterized in plants, the remobilization of Mo between different tissues is much less understood. In *Arabidopsis thaliana*, AtMOT1;2 has been found to regulate the distribution of Mo in different tissues and organs (Gasber et al., 2011). T-DNA insertion mutants of *AtMOT1;2* accumulated more Mo in older leaves but less Mo in seeds than the wild type. Recently, OsMOT1;2, the homolog of *Arabidopsis* AtMOT1;2, was shown to be involved in controlling the accumulation of Mo in rice grains (Ishikawa et al., 2021). A rice mutant harboring a complete deletion of *OsMOT1;2* accumulated significantly lower Mo levels in grains but higher Mo levels in the second and lower leaf sheaths, nodes, and internodes. However, the molybdate transport activity of OsMOT1;2 has not been determined, and its detailed roles remain unclear.

In this study, we investigated the molybdate transport activity of OsMOT1;2 and showed that OsMOT1;2 functions as a vacuolar molybdate exporter that plays an important role in the root-to-shoot translocation of Mo and remobilization of Mo from flag leaves to rice grains. Knockout of *OsMOT1;2* leads to more Mo accumulation in roots and older tissues but less Mo translocation to shoots and grains. Furthermore, the remobilization of Mo from older leaves to young leaves under Mo-depleted condition significantly decreased in the *osmot1;2* mutants. In contrast, overexpression of *OsMOT1;2* enhances the root-to-shoot translocation of Mo and increases the Mo concentration in rice grains. Our work sheds light on the regulation of Mo homeostasis in plant cells and provides a theoretical basis for breeding rice varieties with Mo enriched in grains.

## Materials and Methods

### Plant Materials and Growth Conditions

The rice (*Oryza sativa* L.) cultivar Zhonghua11 (ZH11, *japonica*) was used in this study as the wild type (WT). The knockout mutants of *OsMOT1;2* were generated by CRISPR/Cas9-based genome editing technology. The *OsMOT1;2* overexpression lines were generated by expressing *OsMOT1;2* in ZH11 under the control of a maize ubiquitin promoter.

To study the phenotype of *osmot1;2* at the seedling stage, seeds of the WT and *osmot1;2* were soaked and germinated in an incubator at 37°C for 3 days and then sowed on a plastic net floating with ultra-pure deionized water. After 7 days, the seedlings were transplanted in plastic boxes containing half-strength Kimura B solution (Huang et al., 2016) with or without 10 nM Mo (pH 5.6). The nutrient solution was renewed twice a week. All hydroponic experiments were carried out in a light incubator with a 12-h-light (28°C)/12-h dark (25°C) photoperiod and 60% relative humidity. For analysis of the phenotype of *osmot1;2* at the maturity stage, 4-week-old seedlings of the WT and *osmot1;2* were transplanted in a paddy field. All lines were planted adjacent to each other with 12 replicates, and the agronomic management measures, including water and fertilizer, were the same as those in normal fields. In addition, 2-week-old seedlings of ZH11 were transplanted in a paddy field in Nanjing, Jiangsu, China, for studying the expression of *OsMOT1;2* during the whole growth period.

### Tissue Elemental Analysis

The elemental concentrations in different tissues of rice plants or yeast cells were determined by an inductively coupled plasma mass spectrometer (ICP-MS) (NexION 300D; PerkinElmer Corp., Norwalk, CT, United States) according to previous studies (Huang et al., 2016, 2019). The roots and different leaves of WT, *osmot1;2*, and *OsMOT1;2* overexpression lines grown hydroponically and different tissues at mature growth stages were harvested as described earlier (Huang et al., 2019).

### Vector Constructions and Generation of Transgenic Plants

For the generation of CRISPR/Cas9 knockout constructs, target sequences were selected and primers were designed on the website CRIPSR-GE (Xie et al., 2017). T0 generation of transgenic plants was sequenced to confirm mutation sites in *OsMOT1;2* genomic sequence. The homozygous knockout mutants at T1 or T2 generations were used in the experiments. To study the expression pattern of *OsMOT1;2*, the putative promoter sequence (3,072 bp upstream of the start codon) of *OsMOT1;2* was amplified by PCR using ZH11 genomic DNA as a template. The amplified fragment was cloned into the *Asc*I-*Pac*I site of the PS1aG-3 vector to generate the p*OsMOT1;2*:GUS fusion construct. T1 generation of transgenic plants was identified by hygromycin screening, and the positive lines were used for histochemical GUS staining. To generate the overexpression lines of *OsMOT1;2*, the full-length coding sequence of *OsMOT1;2* (accession number: LOC_Os01g45830) was amplified by PCR using ZH11 cDNA as a template. The full-length coding sequence of eGFP without the stop codon was amplified from the pUN1301-eGFP plasmid. These two fragments were cloned into the *Bam*HI-*Sac*I site of pUN1301-eGFP with the original GFP sequence removed. The positive T1 generation of transgenic plants was identified by histochemical GUS staining, and the expression level of *OsMOT1;2* was determined by qRT-PCR. The constructed vectors above were introduced into the rice calli by *Agrobacterium*-mediated genetic transformation. The primer sequences used for vector construction and genotyping are listed in Supplementary Table 1.

### RNA Extraction, cDNA Synthesis, and Quantitative Reverse Transcription PCR

To study the expression pattern of *OsMOT1;2* during the whole growth period of rice, different tissues and organs of ZH11 were sampled at seedling, tillering, booting, flowering, and grain filling stages. To investigate the expression pattern of *OsMOT1;2* under various nutrient-deficient conditions, 1-week-old ZH11 seedlings cultured with half-strength Kimura B nutrient solution were treated with indicated element omitted nutrient solution for another 1 week. Shoots and roots of the seedlings were harvested separately. Samples were frozen in liquid nitrogen and ground into powder using a grinder or mortar. RNA was extracted by a Universal Plant Total RNA Extraction Kit (BioTeke, Wuxi, China). After removing residual DNA contamination, RNA was reverse transcribed into cDNA using a HiScript II First-Strand cDNA Synthesis Kit (Vazyme, Nanjing, Jiangsu, China). qRT-PCR was performed to determine the relative expression level using 2 × AceQ qPCR SYBR Green Master Mix (Vazyme, Nanjing, Jiangsu, China).

### Functional Analysis of *OsMOT1;2* in Yeast

*OsMOT1;2* was heterologously expressed in yeast (*Saccharomyces cerevisiae*) to determine the molybdate transport activity. The full-length coding sequences of *OsMOT1;2*, *OsMOT1;1*, and *AtMOT1;2* were amplified by PCR using ZH11 or *Arabidopsis* accession Col-0 cDNA as templates. The amplified fragments were cloned into the *Eco*RI-*Xho*I site of pDR196 plasmid to generate a yeast expression vector driven by a constitutive PMA promoter. The constructs and the empty vector were transformed into yeast strain 31019b (MATa ura3 mep1Δ mep2Δ:LEU2 mep3Δ: KanMX2) using a Frozen-EZ Yeast Transformation II Kit (ZYMO Research, Irvine, CA, United States). A single colony of the transformed yeast strains was selected and inoculated overnight (30°C, 200 rpm) in 2 ml SD/-U liquid media without Mo; 1 ml of overnight yeast media was transferred to 50 ml SD/-U liquid media without Mo and incubated at 30°C with 200 rpm until the logarithmic growth period. At this time, Na_2_MoO_4_ was added to the media to a final concentration of 0.5 μM, and the solution was incubated at 30°C for 30 min. Yeast cells were harvested by centrifugation at 4°C and washed three times with precooled EDTA-Na_2_ solution and once with precooled Milli-Q water. Yeast cells were dried, weighed, and then digested to determine Mo concentration by ICP-MS as described earlier.

To investigate whether OsMOT1;2 has sulfate transport activity, the full-length coding sequence of *OsMOT1;2* was cloned into the *Eco*RI-*Xho*I site of pYX222x to generate a yeast expression vector driven by a constitutive TPI promoter. The fused vector and empty vector were transformed into a sulfate uptake defect yeast mutant CP154-7B (MATa, his3, leu2, ura3, ade2, trp1, sul1:LEU2, sul2:URA3) (Tomatsu et al., 2007; Huang et al., 2019). *AtSULTR1;2*, which encodes an *Arabidopsis thaliana* high-affinity sulfate transporter, was used as the positive control. The overnight cultures of yeast cells were collected and washed three times with Milli-Q water. The OD_600_ of yeast cultures was adjusted to 1.0, and then, 4 μl yeast series dilution suspensions were spotted on synthetic media containing 0.5 mM sulfate with or without methionine (Met). The plates were incubated at 30°C for 4 days before taking pictures.

### Subcellular Localization of *OsMOT1;2*

To investigate the subcellular localization of OsMOT1;2, the full-length coding sequence of *OsMOT1;2* was amplified by PCR using ZH11 cDNA as a template. The amplified fragment was cloned into the *Xba*I-*Bam*HI site of pCAMBIA1301-A7-GFP vector to generate the *35S:GFP-OsMOT1;2* construct. The *35S:GFP-OsMOT1;2* construct was transformed into *Agrobacterium tumefaciens* strain GV3101. The *pSY06-SV40-NLS-mCherry* construct, which expresses a nuclear localization mCherry protein, was also transformed into *A. tumefaciens* strain GV3101 to indicate the nuclei. The positive strains of these two constructs were identified by colony PCR and then co-injected into *Nicotiana benthamiana* leaves. The fluorescence of GFP and mCherry was observed using a laser confocal microscope (TCS SP8 X, Leica, Germany).

### Microfocus X-ray Fluorescence (μ-XRF)

To determine the accumulation of Mo in grains of WT and *osmot1;2*, seeds were manually dehusked and attached to a Kapton tape using a plastic tweezer and then scanned on an X-ray fluorescence (μ-XRF) spectrometer (M4 TORNADO PLUS, Bruker, Germany). The relative fluorescence intensities of μ-XRF images were quantified using ImageJ v1.8.0^1^.

### Phylogenetic Analysis and Structure Prediction

To obtain the protein sequences of MOT1 family proteins, the protein sequence of OsMOT1;1 was used as a query sequence to blast the NCBI or Gramene databases. The phylogenetic tree was constructed using MEGA7, and conserved motif prediction was carried out on the website MEME Suite.^2^ The phylogenetic tree and conserved motif were visualized in the TBtools software (Chen et al., 2020). The transmembrane domain prediction of OsMOT1;1 and OsMOT1;2 were performed using the ARAMEMNON 8.1^3^ with up to 18 individual programs. The individual predictions of OsMOT1;1 or OsMOT1;2 were combined to a built-in consensus prediction. The consensus diagram only included the transmembrane segments with consensus scores equal to or above 0.42 according to the instructions of ARAMEMNON. The secondary topology images of OsMOT1;1 and OsMOT1;2 were created by the TOPO2 software^4^ and polished by the Protter tool.^5^ The three-dimensional structures of OsMOT1;1 and OsMOT1;2 were retrieved from AlphaFold Protein Structure Database^6^.

## Results

### Identification of *OsMOT1;2* in the Rice Genome and Structure Prediction

To identify OsMOT1;2 in rice, the protein sequence of CrMOT1, the first MOT1 protein isolated from green alga *C. reinhardtii* (Tejada-Jimenez et al., 2007), was used as a prey to search for the MOT1 homologs in the rice genome. Two MOT1 proteins were identified and named as OsMOT1;1 and OsMOT1;2 according to the phylogenesis of MOT1 proteins from several plants, including rice (*Oryza sativa*), *Arabidopsis thaliana*, maize (*Zea mays*), legume (*Lotus japonicus*), and *Medicago truncatula* (Supplementary Figure 1A). The MOT1 proteins generally have five conserved motifs (Supplementary Figure 1A), including the PXPVQPMKX(I/L)(A/G)AXA motif and the FGXMPXCHG(S/A)GGLAXQ(Y/H)XFG(A/G)RXG motif, which have been identified earlier (Tejada-Jimenez et al., 2007). To better understand the secondary structures of OsMOT1;1 and OsMOT1;2, we predicted the transmembrane domains of these two proteins by using up to 18 individual prediction programs (Supplementary Figures 1B–E). The consensus predictions suggested that OsMOT1;1 and OsMOT1;2 have 9 and 10 transmembrane domains, respectively (Supplementary Figures 1B,C). The N-terminal of OsMOT1;1 was predicted to localize to the extracellular compartment, while the N-terminal of OsMOT1;2 was present in the cytoplasm (Supplementary Figures 1B,C). We also used AlphaFold, a novel machine learning approach that incorporates physical and biological knowledge about protein structure (Jumper et al., 2021; Varadi et al., 2021), to predict the three-dimensional structure of OsMOT1;1 and OsMOT1;2. In contrast to 9 and 10 transmembrane domains of OsMOT1;1 and OsMOT1;2 predicted by traditional protein structure prediction programs, AlphaFold predicts that both proteins have 14 transmembrane domains with the last two domains at the C-terminal not fully integrated into the membrane (Supplementary Figures 1F–G).

### OsMOT1;2 Exhibits Molybdate Transport Activity

**FIGURE 1:**
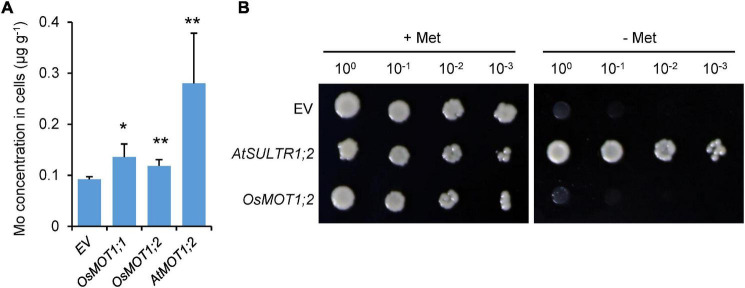
Functional analysis of *OsMOT1;2* in yeast. **(A)** Molybdate transport activity of OsMOT1;2 in yeast. Yeast cells transformed with empty vector (EV), *OsMOT1;1, OsMOT1;2*, or *AtMOT1;2* were incubated in the media containing 0.5 μM Mo for 30 min. Mo concentrations in yeast cells were determined. Data are presented as means ± SD with three biological replicates. * and ** represent significant differences at *P* < 0.05 and *P* < 0.01, respectively (Student’s *t*-test). **(B)** Sulfate transport activity of OsMOT1;2 in yeast. The yeast mutant strain CP154-7B transformed with empty vector (EV), *AtSULTR1;2*, or *OsMOT1;2* were serially diluted and grown on the media containing with or without added methionine (Met) for 3 days.

Because OsMOT1;1 has been shown to control Mo accumulation in rice grains (Yang et al., 2018; Huang et al., 2019; Wang et al., 2020), we focused on OsMOT1;2 in this study. To test whether OsMOT1;2 exhibits molybdate transport activity, *OsMOT1;2* was heterologously expressed in yeast (*Saccharomyces cerevisiae*) strain BY4741. *OsMOT1;1* was used as a positive control (Huang et al., 2019). Yeast cells transformed with empty vector, *OsMOT1;1*, or *OsMOT1;2* were cultured in a Mo-free medium to the mid-log phase and then transferred to a medium containing 0.5 μM Mo. As shown in Figure 1A, the Mo concentrations in yeast cells transformed with *OsMOT1;1* or *OsMOT1;2* were significantly higher than the control strain transformed with an empty vector (Figure 1A). These results indicated that similar to OsMOT1;1, OsMOT1;2 can transport molybdate. We further determined the molybdate transport activity of *Arabidopsis* AtMOT1;2. Yeast cells transformed with *AtMOT1;2* accumulated a significantly higher level of Mo in the cells compared with the empty vector control (Figure 1A), suggesting AtMOT1;2 also exhibits the molybdate transporting activity. The Mo concentration in the yeast cells transformed with *AtMOT1;2* was more than twofold higher than the yeast strains transformed with *OsMOT1;1* or *OsMOT1;2* (Figure 1A), indicating that OsMOT1;2 exhibits higher molybdate transport activity than OsMOT1;1 or OsMOT1;2.

MOT1 family proteins share sequence similarity to sulfate transporters and were previously considered as group five of the sulfate transporter family (Kumar et al., 2011). Previous studies have confirmed that OsMOT1;1 and AtMOT1;1 lack the sulfate transport activity (Tomatsu et al., 2007; Huang et al., 2019). To determine whether OsMOT1;2 exhibits a sulfate transport activity, we performed complementation analysis in yeast. The yeast mutant CP154-7B is defective in two high-affinity sulfate transporters and could not grow on low sulfate media without methionine (-Met media) (Shibagaki et al., 2002; Yoshimoto et al., 2002; Tomatsu et al., 2007). The yeast mutant transformed with an *A. thaliana* high-affinity sulfate transporter gene, *SULTR1;2*, was able to grow on the –Met media (Figure 1B). However, similar to the empty vector, the expression of *OsMOT1;2* in the yeast mutant was unable to restore the growth of the mutant strain on –Met media (Figure 1B). These results suggest that OsMOT1;2 lacks the sulfate transport activity.

### Expression Pattern of *OsMOT1;2*

**FIGURE 2:**
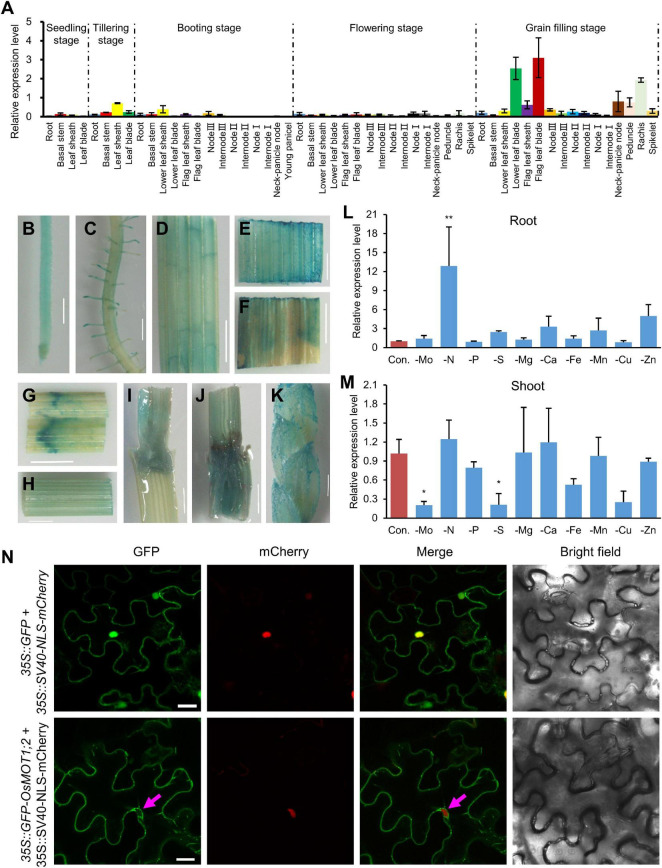
Expression pattern and subcellular localization of OsMOT1;2. **(A)** Relative expression level of *OsMOT1;2* in different tissues of ZH11 at different growth stages. Samples were collected from the plants grown in a paddy field, and the relative expression level was determined by qRT-PCR. The expression level of *OsMOT1;2* was normalized to the housekeeping gene *OsActin* and shown as 2^–ΔCt^. **(B–K)** Histochemical GUS (β-glucuronidase) staining of different tissues of transgenic plants transformed with the GUS construct driven by the *OsMOT1;2* promoter. GUS staining was performed in the root **(B,C)**, blade **(D)**, and sheath **(E)** of 14-day-old transgenic plants grown hydroponically, and the flag leaf blade **(F)**, flag leaf sheath **(G)**, internode 2 **(H)**, node 2 **(I)**, node 3 **(J)**, and the spikelet **(K)** of transgenic plants grown in potting soils at the grain filling stage. Bar, 0.5 cm. **(L,M)** Expressions of *OsMOT1;2* under different nutrient deficiencies. Two-week-old plants were treated for 1 week in the nutrient solution with different nutrient depletions. Expressions of *OsMOT1;2* were determined in roots **(L)** and shoots **(M)** by qRT-PCR. **(N)** Subcellular localization of OsMOT1;2. OsMOT1;2 was fused with green fluorescence protein (GFP) at N-terminus and expressed in tobacco leaves by a cauliflower mosaic virus 35S promoter. The 35S:SV40-NLS-mCherry was co-expressed to show the location of the nucleus. The magenta arrows show the GFP fluorescence in the inner side of the nucleus. Bar, 20 μm. Data in **(A,L,M)** are presented as means ± SD with three biological replicates. Columns with * or ** in **(L,M)** indicate significant differences between the treatment and the control at *P* < 0.01 or *P* < 0.05 (Dunnett’s multiple comparison test).

The tissue expression pattern of *OsMOT1;2* was determined by qRT-PCR in various tissues of plants grown in a paddy field at different growth stages during the whole growth period. *OsMOT1;2* was strongly expressed in the blades of the flag leaf and the second leaf during the grain filling stage (Figure 2A). The expression of *OsMOT1;2* was also generally high in the sheath, panicle node, peduncle, and rachis at the grain filling stage (Figure 2A). However, the expression of *OsMOT1;2* was much lower in most tissues of field-grown plants at other growth stages, including the seedling stage, tillering stage, booting stage, and flowering stage (Figure 2A). The expression pattern of *OsMOT1;2* was further determined in the *OsMOT1;2* promoter-GUS transgenic rice plants. In the hydroponically grown transgenic seedlings, GUS signals were detected in the root, blade, and sheath (Figures 2B–E). At the grain filling stage, GUS signals were detected in the blade, sheath, node, and spikelet (Figures 2F–K).

We further determined the expression of *OsMOT1;2* in response to the depletion of Mo or other mineral nutrients, including N, P, S, Mg, Ca, Fe, Mn, Cu, or Zn. Two-week-old plants grown in the half-strength Kimura nutrient were transferred to the nutrient solution without the above mineral nutrients and were grown for a further week. The expression of *OsMOT1;2* in shoots was suppressed by Mo depletion but such suppression was not found in roots (Figures 2L,M). In roots, *OsMOT1;2* was strongly induced by N deficiency and the deficiencies of S and Zn to a less extent. However, *OsMOT1;2* was downregulated under S deficiency in shoots but no significant change was observed under Zn deficiency. The expression of *OsMOT1;2* was also suppressed by Fe and Cu deficiencies in shoots (Figures 2L,M).

### OsMOT1;2 Localizes to Tonoplast

To determine the subcellular localization, OsMOT1;2 fusion with GFP at the N-terminal was transiently expressed in tobacco (*Nicotiana benthamiana*) leaves under the control of a cauliflower mosaic virus 35S promoter (*35S:GFP-OsMOT1;2*). A *35S:SV40-NLS-mCherry* construct in which the mCherry was fused with the nuclear localization sequence (NLS) of SV40 was co-expressed to show the nuclei. For the control construct (*35S:GFP*), the GFP signals were observed in the whole cells, including the nuclei and the plasma membrane (Figure 2N). In contrast, the GFP signals in the tobacco leaves expressing the *35S:GFP-OsMOT1;2* construct were found on the vacuolar membrane as the signals were observed on the inside facing side of the nuclei (Figure 2N). These results indicated that OsMOT1;2 localizes to the tonoplast.

### Knockout of *OsMOT1;2* Decreases the Root-to-Shoot Translocation of Molybdenum

**FIGURE 3:**
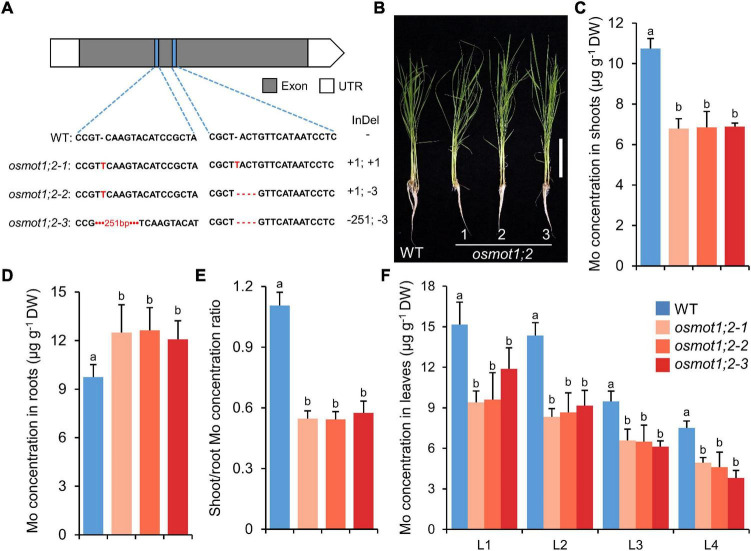
Knockout of *OsMOT1;2* reduces the root-to-shoot translocation of Mo. **(A)** Schematic diagram of three knockout lines of *OsMOT1;2*. Blue rectangles represented the sgRNA target sites on the *OsMOT1;2* exon, and the sequences are shown below. Nucleotide deletion is indicated by red dashes; short and long insertions are indicated by red letter and red number, respectively. **(B)** The growth phenotype of WT and three knockout lines of *osmot1;2* cultured with nutrient solution with 10 nM Mo at four leaves seedling stage. Vertical bar, 10 cm. **(C–E)** The Mo concentrations in shoots **(C)**, roots **(D)**, and the shoot/root concentration ratio **(E)** of the seedlings are described in **(B)**. **(F)** The Mo concentrations in different leaves of the seedlings in **(B)**. L1, L2, L3, and L4 represent leaves at different positions. Data in **(C–F)** are presented as means ± SD with five or six biological replicates. Columns with different letters in **(C–F)** indicate significant differences at *P* < 0.05 (Tukey’s honestly significant difference test).

To investigate the functions of *OsMOT1;2* in rice, we generated the knockout mutants by CRISPR/Cas9 genome editing system. Three independent knockout lines were generated with either the coding frame shifted or sequence deletions at two different target sites in the coding region of *OsMOT1;2* (Figure 3A). The knockout lines had no obvious growth differences from the wild type (WT) under hydroponic growth conditions (Figure 3B and Supplementary Figure 2). In the shoots of 4-week-old knockout lines grown in the nutrient solution containing 10 nM Mo, the Mo concentrations decreased by 35.9–36.9% compared with the WT (Figure 3C). However, the Mo concentrations in roots were increased by 23.5–29.3% (Figure 3D). By calculation of the shoot/root concentration ratio, the *osmot1;2* knockout mutants had a significantly lower Mo translocation ratio compared with the WT (Figure 3E). We further determined the Mo concentrations in different leaves and found that the Mo concentrations in all four leaves of *osmot1;2* were significantly lower than those of the WT (Figure 3F).

Among the 9 other mineral elements determined, the concentrations of P and Zn were significantly increased in the shoots and roots of all three knockout lines (Supplementary Figures 3A,B). The concentration of Cu was significantly increased in the roots of two knockout lines, while no consistent difference was found for the concentrations of B, S, K, Ca, Mn, and Fe in either shoots or roots (Supplementary Figures 3A,B). Although the concentrations of P and Zn increased in both shoots and roots, the translocation ratios of P and Zn from roots to shoots were comparable with the WT (Supplementary Figure 4). However, the translocation ratio of Cu was significantly decreased in all three knockout lines (Supplementary Figure 4).

### Alterations of Molybdenum Remobilization in *Osmot1;2* at the Seedling Stage

**FIGURE 4:**
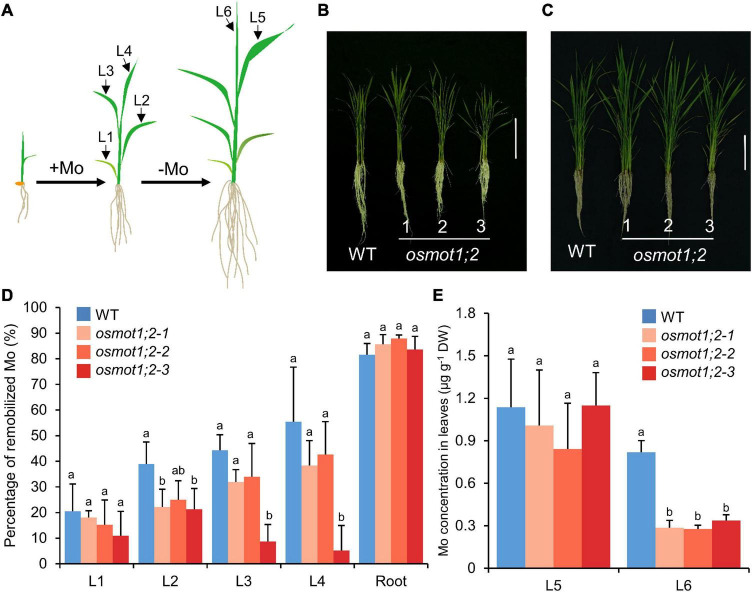
Knockout of *OsMOT1;2* decreases the remobilization of Mo from older leaves to young leaves under Mo-depleted condition. **(A)** Schematic diagram showing the experimental setup. WT and three knockout lines of *osmot1;2* plants were grown in nutrient solution with 10 nM Mo until the fourth leaf was fully expanded. Plants were then transferred to the nutrient solution without adding Mo and were grown to six leaves stage. **(B,C)** Images of WT and three knockout lines of *osmot1;2* at four leaves stage **(B)** and six leaves stage **(C)**. Vertical bar, 10 cm. **(D)** The percentage of Mo in roots and the first to fourth leaves (L1–L4) that were remobilized to new tissues. The percentage of remobilized Mo in roots or corresponding leaves was calculated by the difference of Mo concentrations between the four leaves stage and six leaves stage and divided by the concentrations at the four leaves stage. **(E)** The Mo concentrations in the fifth and sixth leaves (L5 and L6) at the six leaves stage were newly grown after Mo-depleted treatment. Data in **(D,E)** are presented as means ± SD with five or six biological replicates. Columns with different letters in **(D,E)** indicate significant differences at *P* < 0.05 (Tukey’s honestly significant difference test).

To investigate whether the mutation of *OsMOT1;2* affects the distribution of Mo among different tissues, we determined the Mo remobilization from old leaves to new leaves after Mo shortage treatment. Plants were grown in a nutrient solution containing 10 nM Mo to 4-leaf-old stage, and then, the Mo supply in the nutrient solution was withdrawn until the sixth leaf was fully expanded (Figures 4A–C). The differences in Mo concentrations in roots and each leaf between the four- and six-leaf-old stages were determined and used to calculate the percentage of Mo remobilization. In roots, 81.5% of Mo was remobilized in WT, while 83.6–87.9% of Mo was remobilized in *osmot1;2* (Figure 4D). The remobilization of Mo in leaves was significantly lower in *osmot1;2* compared with that of WT, particularly in the second (L2, 21.3–25.0 vs. 38.9%; *P* < 0.05), third (L3, 8.7–34.0 vs. 44.3%), and fourth leaves (L4, 5.1–42.7 vs. 55.5%) (Figure 4D). Consistent with smaller Mo remobilization from old leaves, the Mo concentration in the newly grown sixth leaf was lower in *osmot1;2* than that of WT (Figure 4E). These results suggest that OsMOT1;2 is involved in the remobilization of Mo from old leaves to young leaves.

### The *Osmot1;2* Mutant Accumulates More Molybdenum in Lower Nodes but Less Molybdenum in Grains

**FIGURE 5:**
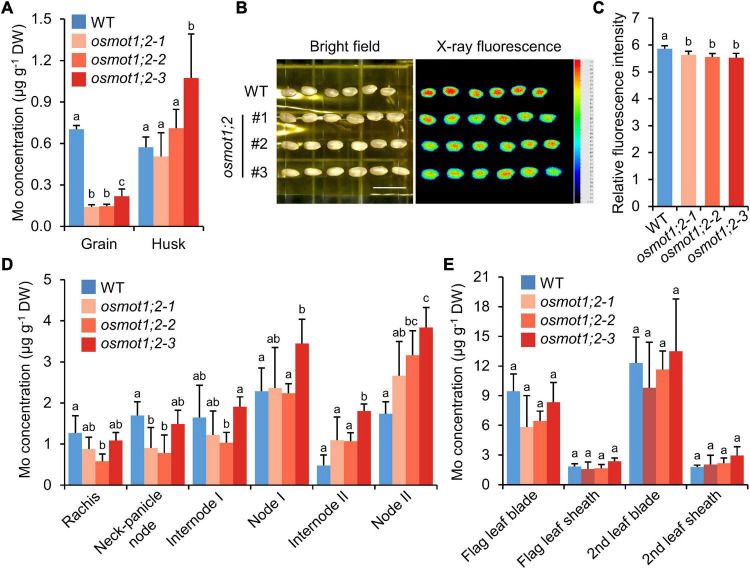
Knockout of *OsMOT1;2* alters Mo allocation among various tissues at harvesting stage. **(A)** The Mo concentration in grains and husks of WT and *osmot1;2*. **(B)** The images of μ-XRF of grains of WT and *osmot1;2*. Left: the image at bright filed; right: the image scanned by μ-XRF. **(C)** Relative fluorescence intensity of μ-XRF images shown in **(B)**. **(D,E)** The Mo concentrations in different tissues of WT and *osmot1;2* at mature stage. Data in **(A,C–E)** are presented as means ± SD with five or six biological replicates. Columns with different letters in **(A,C–E)** indicate significant differences at *P* < 0.05 (Tukey’s honestly significant difference test).

To explore whether the knockout of *OsMOT1;2* affects Mo accumulation in the grains, WT and *osmot1;2* were grown in a paddy field until maturity. Compared with the WT, the Mo concentration in grains of *osmot1;2* was 69.0–79.9% lower (Figure 5A). Among the 18 elements determined, the concentration of rubidium (Rb) was also significantly decreased in the grains of *osmot1;2* (Supplementary Figure 5). However, the concentrations of Cu and Ni were significantly increased (Supplementary Figure 5).

The Mo level in grains of *osmot1;2* was further determined by μ-XRF. Compared with the WT, *osmot1;2* showed lower Mo fluorescence intensity in the μ-XRF images (Figures 5B,C). These results were consistent with lower Mo concentration in grains of *osmot1;2* as determined by ICP-MS (Figure 5A). There was no consistent difference in the Mo concentration between *osmot1;2* and WT in husk, leaf blade, and leaf sheath of the flag leaf and second leaf counting from the top of plants, while the Mo concentrations in internode 2 and node 2 of *osmot1;2* were significantly higher than those of WT (Figures 5A,D,E). In addition, there is a trend that *osmot1;2* accumulated less Mo in the upper part of the plants, including rachis and neck-panicle node (Figure 5D). These results suggest that OsMOT1;2 regulates Mo allocation in different tissues of mature plants.

### Knockout of *OsMOT1;2* Has No Effect on Agronomic Traits

To investigate the effect of knockout of *OsMOT1;2* on the agronomic traits of rice, the plants of WT and *osmot1;2* were grown in the field until maturity, and main agronomic traits, including plant height, effective panicles per plant, grains number per panicle, seed setting rate, yield of a single plant, and 1,000-grain weight, were surveyed. No obvious difference was found in growth status (Supplementary Figure 6A) nor in the surveyed traits (Supplementary Figures 6B–G) between WT and *osmot1;2*, although a slight decrease in the seed setting rate occurred in one line of *osmot1;2* (Supplementary Figure 6E). These results indicated that the deletion of *OsMOT1;2* has no effect on the agronomic traits of rice.

### Overexpression of *OsMOT1;2* Enhances the Root-to-Shoot Translocation of Molybdenum

**FIGURE 6:**
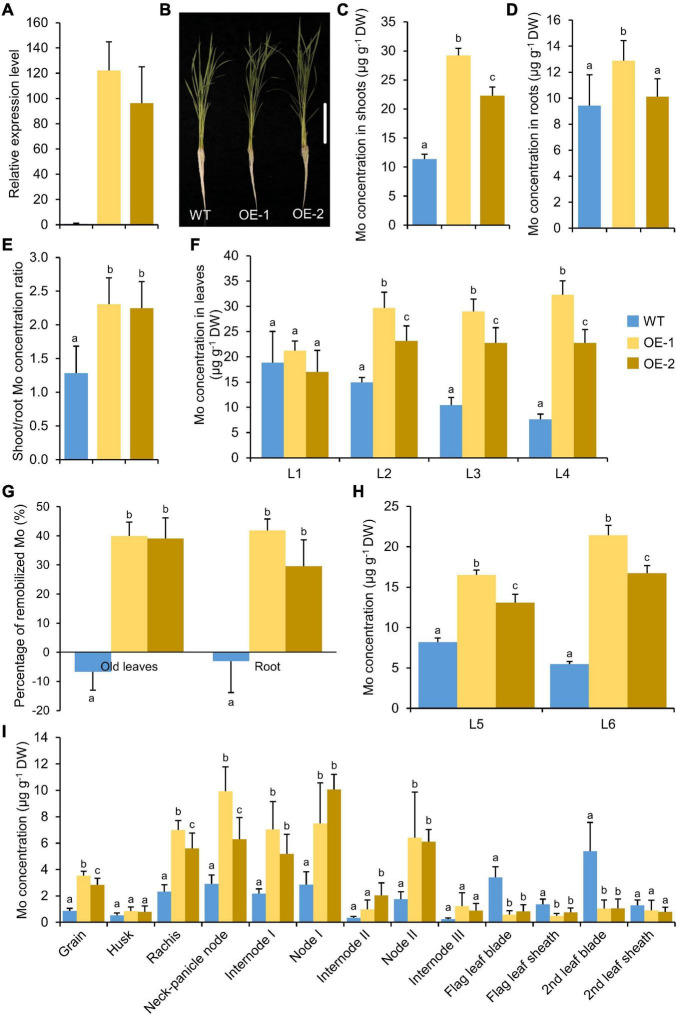
Overexpression of *OsMOT1;2* enhances root-to-shoot translocation and Mo remobilization. **(A)** Relative expression level of *OsMOT1;2* in WT and *OsMOT1;2* overexpression lines. The relative expression level of *OsMOT1;2* was determined by qRT-PCR and was normalized to the housekeeping gene *OsActin* and shown as 2^–ΔCt^. **(B)** The growth phenotype of WT and two *OsMOT1;2* overexpression lines cultured with nutrient solution with 10 nM Mo at four leaves seedling stage. Vertical bar, 10 cm. **(C–E)** The Mo concentrations in shoots **(C)**, roots **(D)**, and the shoot/root concentration ratio **(E)** of the seedlings described in **(B)**. **(F)** The Mo concentrations in different leaves of the seedlings in **(B)**. L1, L2, L3, and L4 represent the first to fourth leaves. **(G)** The percentage of Mo in roots and older leaves (the first to fourth leaves; L1–L4) that were remobilized to new leaves (the fifth and sixth leaves; L5 and L6). The percentage of remobilized Mo in roots or old leaves was calculated by the difference of Mo concentrations between the four leaves stage and six leaves stage and divided by the concentrations at the four leaves stage. **(H)** The Mo concentrations in the fifth and sixth leaves (L5 and L6) at the six leaves stage were newly grown after Mo-depleted treatment. **(I)** The Mo concentrations in various tissues of WT and two independent *OsMOT1;2* overexpression lines (OE-1 and OE-2). Data in **(A,C–H)** are presented as means ± SD with three **(A)** or six **(C–I)** biological replicates. Columns with different letters in **(A,C–H)** indicate significant differences at *P* < 0.05 (Tukey’s honestly significant difference test).

To determine whether the overexpression of *OsMOT1;2* could change the allocation of Mo between different tissues, *OsMOT1;2* was overexpressed under the control of rice ubiquitin promoter. Two independent overexpression lines with 90–120 times higher expression of *OsMOT1;2* were chosen for experiments (Figure 6A). The overexpression lines showed no obvious changes in growth phenotype compared with the WT (Figure 6B and Supplementary Figures 2C,D). The overexpression lines accumulated a significantly higher level of Mo in shoots compared with that of WT (Figure 6C). However, the Mo concentrations were not consistently changed in roots (Figure 6D). The shoot/root Mo concentration ratio of the overexpression lines was 1.9-fold higher compared with the WT (Figure 6E), suggesting that overexpression of *OsMOT1;2* enhanced the translocation of Mo from roots to shoots. The elevation of Mo concentration in the overexpression lines was stronger in younger leaves with an average of 2.6-, 1.5-, and 0.8-fold higher in the fourth, third, and second leaves, respectively, compared with the corresponding leaves of WT (Figure 6F). However, there was no change in the first leaves.

The concentrations of several elements were also changed in the shoots and roots of the overexpression lines. Mn and Zn concentrations were significantly increased in the shoots, while the concentrations of S, K, and Cu increased in roots (Supplementary Figure 7). Only the shoot/roots ratio of S, K, and Cu was significantly decreased in the overexpression lines (Supplementary Figure 8).

The translocation of Mo from roots or old leaves to newly grown leaves after Mo depletion treatment was determined. In the overexpression lines, the translocation rate of Mo from roots and old leaves (leaves L2–L4) to newly grown leaves (leaves L5 and L6) was significantly higher than that of the WT (Figure 6G). Consistent with the enhancement of Mo remobilization from roots and old leaves, the Mo concentrations in newly grown leaves L5 and L6 were significantly higher than that of the WT (Figure 6H).

### Overexpression of *OsMOT1;2* Increases the Accumulation of Molybdenum in Grains and Nodes

Overexpression of *OsMOT1;2* enhanced the root-to-shoot translocation of Mo at the seedling stage (Figure 6E). We further investigated whether the overexpression of *OsMOT1;2* affects the translocation of Mo among different tissues, particularly the Mo accumulation in grains. Two independent overexpression lines and corresponding WT were grown in potting soil until maturity. The Mo concentrations in the grains and the first and second nodes and internodes were all significantly higher than those of the WT (Figure 6I). In contrast, the Mo concentrations in the blades and sheaths connecting the first and second nodes, namely the flag leaf and the second leaf, respectively, were all lower than those of the WT (Figure 6I). The results indicated that overexpression of *OsMOT1;2* enhanced the translocation of Mo from leaves to nodes and grains during grain filling. Also, the concentrations of Cu and S were significantly lower in the grains of *OsMOT1;2* overexpression lines, while no consistent changes were found in other elements (Supplementary Figure 9).

## Discussion

Although the mechanism of Mo uptake and transport in plants has been preliminarily understood at present, it is still of great value to explore the molecular mechanisms underlying Mo accumulation in plants due to the important roles of Mo in plant growth and human health. In this study, we characterized the functions of OsMOT1;2, a member of MOT1 family in rice, using *in vivo* and *in vitro* experiments. Heterologous expression of *OsMOT1;2* in yeast showed that OsMOT1;2 has Mo transport activity (Figure 1A). Subcellular localization revealed that OsMOT1;2 localizes to tonoplast. Knockout of *OsMOT1;2* led to an increase of Mo concentration in roots but a decrease of Mo concentration in shoots at seedlings stage (Figures 3C,D), and less Mo accumulation in grains at harvesting stage (Figures 5A–C). In contrast, overexpression of *OsMOT1;2* enhances the translocation of Mo from roots to shoots at seedling stage (Figures 6C–E), and from leaves to grains at mature stage (Figure 6I). Our results suggest that OsMOT1;2 functions as a vacuolar molybdate exporter and plays important roles in controlling cellular Mo homeostasis and allocation of Mo among different tissues in rice.

In *Arabidopsis*, AtMOT1;2 was responsible for the interorgan translocation and allocation of Mo (Gasber et al., 2011). Recently, OsMOT1;2 has also been shown to participate in the interorgan molybdate distribution in rice (Ishikawa et al., 2021). However, both studies did not detect their molybdate transport activities. In this study, by heterologous expression of *OsMOT1;2* in yeast, we were able to determine the molybdate transport activities of both OsMOT1;2 and AtMOT1;2 (Figure 1A). The molybdate transport activity of OsMOT1;2 was similar to that of OsMOT1;1 (Figure 1A), which has previously been shown to transport molybdate in yeast (Huang et al., 2019). The molybdate transport activity of AtMOT1;2 appeared to be higher than that of OsMOT1;1 and OsMOT1;2 (Figure 1A). The members of MOT1 family were previously considered to belong to group V of the sulfate transporter family (Kumar et al., 2011). However, OsMOT1;2 lacks sulfate transport activity as determined by the heterologous expression in yeast (Figure 2B), similar to that of OsMOT1;1 and AtMOT1;1 (Tomatsu et al., 2007; Huang et al., 2019). Therefore, the MOT1 family proteins in plants are likely molybdate-specific transporters.

The translocation of mineral nutrients from roots to shoots and remobilization from older tissues to young tissue are essential for maintaining plant growth and development, particularly under nutrient-limited conditions. Given the tonoplast localization of OsMOT1;2 (Figure 2N), we hypothesized that OsMOT1;2 may function as a vacuolar molybdate exporter and mediate the remobilization of Mo among different tissues in rice. This hypothesis is supported by several lines of evidence. First, knockout of *OsMOT1;2* resulted in more Mo accumulation in roots and less Mo translocation to shoots (Figures 3C,D), whereas overexpression of OsMOT1;2 enhances the root-to-shoot translocation of Mo (Figures 6C–E). Second, *osmot1;2* accumulated more Mo in lower nodes and internodes but less Mo in grains at mature stage (Figures 5A–D). In contrast, overexpression of *OsMOT1;2* leads to more Mo mobilization from leaves and sheaths to nodes and upper parts of plants, such as grains and rachis (Figure 6I). More Mo accumulation in roots or lower nodes and internodes could be explained by the efflux of less Mo from vacuole for remobilization in the *osmot1;1* mutant. Similarly, a lower level of Mo in the roots and leaves of *OsMOT1;2* overexpression lines is due to the increase of Mo efflux from vacuoles, and more Mo is delivered to sink organs, such as grains.

**FIGURE 7:**
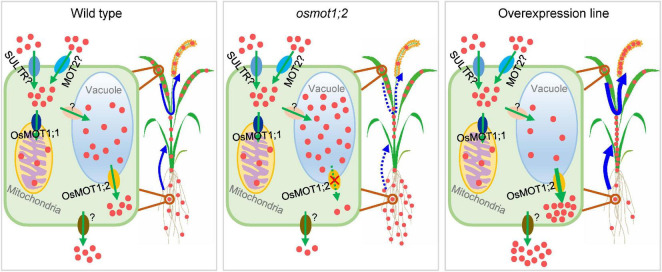
Proposed model of potential roles of OsMOT1;2 in controlling cellular Mo homeostasis and remobilization of Mo in rice. Mo is taken up into cells in the form of molybdate (red dots in the figures) likely through molybdate transporter family 2 protein (MOT2) and/or non-specifically by sulfate transporters (SULTR). Molybdate is then transported into mitochondria by OsMOT1;1 or into vacuoles by unknown transporter(s). OsMOT1;2 functions as a molybdate exporter to mediate the efflux of Mo from vacuoles to the cytosol, which is further transported out of cells for remobilization by unidentified transporter(s). Knockout of *OsMOT1;2* reduces the efflux of Mo from vacuoles, resulting in less Mo being translocated from roots to shoots and from leaves to grains. In contrast, overexpression of *OsMOT1;2* promotes the efflux of Mo from vacuoles and enhances the root-to-shoot translocation and the remobilization of Mo from leaves to grains during filling stage and thus increasing the Mo accumulation in grains.

We thus propose a model in which OsMOT1;2 mediates the cellular Mo homeostasis and allocation among different tissues (Figure 7). Plants take up Mo from soils through both molybdate-specific uptake pathway and non-specific molybdate uptake pathway mainly in the form of molybdate (Huang et al., 2021). In the non-specific molybdate uptake pathway, molybdate could be transported into cells by sulfate transporters due to the similar biochemical properties of molybdate and sulfate (Fitzpatrick et al., 2008). The uptake of molybdate could also be mediated by the major facilitator superfamily protein MOT2. In *C. reinhardtii*, CrMOT2 has been established to be a high-affinity molybdate transporter (Tejada-Jimenez et al., 2011). There are two copies of MOT2 in rice. OsMOT2;1/OsCd1 is involved in root Cd uptake and Cd accumulation in rice grain (Yan et al., 2019). OsMOT2;2/ASY plays an important role in the regulation of early shoot development in rice (Hibara et al., 2013). However, whether OsMOT2;1 and OsMOT2;2 have molybdate transport activities required further studies. Once entry into cells, molybdate is incorporated into the Mo cofactor in the cytosol, serving as the active site of Mo-requiring enzymes (Schwarz and Mendel, 2006; Schwarz et al., 2009). Part of the molybdate could be transported into mitochondria through OsMOT1;1 (Huang et al., 2019) or into vacuoles by unknown transporter(s). The molybdate stored in the vacuole can be effluxed into the cytosol by OsMOT1;2, which could be further transported out of cells and loaded into xylem and/or phloem for translocation to shoots or remobilization to grains (Figure 7). In the *osmot1;2* mutant, more molybdate is sequestered in the vacuole and results in less Mo being translocated to shoots or delivered to grains (Figure 7). In contrast, in the OsMOT1;2 overexpression line, the efflux of Mo from the vacuole is strengthened, which leads to the enhancement of root-to-shoot translocation and the remobilization from leaves to grains (Figure 7). The sulfate transporters SULTR4;1 and SULTR4;2 in *Arabidopsis* have been shown to mediate the efflux of sulfate from vacuole (Kataoka et al., 2004). Whether these two transporters mediate the efflux of molybdate is not clear. Furthermore, the transporters responsible for the influx of molybdate into the vacuole and the efflux of molybdate out of plant cells remain to be identified.

## Conclusion

In conclusion, our work demonstrates that OsMOT1;2 exhibits molybdate transport activity and functions as a vacuolar exporter participating in the root-to-shoot translocation and remobilization of Mo from flag leaves to grains. We also show that overexpressing *OsMOT1;2* can significantly enrich rice grains with Mo, a strategy that could be explored to benefit the nutritional requirements of humans. Furthermore, overexpression of *OsMOT1;2* may be a strategy to improve Mo utilization efficiency in plants in low Mo soils.

## Data Availability Statement

The original contributions presented in the study are included in the article/Supplementary Material, further inquiries can be directed to the corresponding author/s.

## Author Contributions

X-YH designed the research. DH and ML performed the experiments. X-YH and DH analyzed the data. X-YH and DH wrote the manuscript with contributions from F-JZ. All authors contributed to the article and approved the submitted version.

## Conflict of Interest

The authors declare that the research was conducted in the absence of any commercial or financial relationships that could be construed as a potential conflict of interest.

## Publisher’s Note

All claims expressed in this article are solely those of the authors and do not necessarily represent those of their affiliated organizations, or those of the publisher, the editors and the reviewers. Any product that may be evaluated in this article, or claim that may be made by its manufacturer, is not guaranteed or endorsed by the publisher.

## Footnotes

1
https://imagej.nih.gov/ij/

2
https://meme-suite.org/meme/tools/meme

3
http://aramemnon.botanik.uni-koeln.de/index.ep

4
http://www.sacs.ucsf.edu/TOPO2/

5
http://wlab.ethz.ch/protter/start/

6
www.alphafold.ebi.ac.uk

## References

[B1] Arnon D. I. Stout P. R. (1939). Molybdenum as an essential element for higher plants. *Plant Physiol.* 14 599–602. 10.1104/pp.14.3.599 16653589 PMC437771

[B2] Baxter I. Muthukumar B. Park H. C. Buchner P. Lahner B. Danku J. (2008). Variation in molybdenum content across broadly distributed populations of *Arabidopsis thaliana* is controlled by a mitochondrial molybdenum transporter (MOT1). *PLoS Genet.* 4:e1000004. 10.1371/journal.pgen.1000004 18454190 PMC2265440

[B3] Bittner F. (2014). Molybdenum metabolism in plants and crosstalk to iron. *Front. Plant Sci.* 5:28. 10.3389/fpls.2014.00028 24570679 PMC3916724

[B4] Chen C. Chen H. Zhang Y. Thomas H. R. Frank M. H. He Y. (2020). TBtools: an integrative toolkit developed for interactive analyses of big biological data. *Mol. Plant* 13 1194–1202. 10.1016/j.molp.2020.06.009 32585190

[B5] Duan G. Hakoyama T. Kamiya T. Miwa H. Lombardo F. Sato S. (2017). LjMOT1, a high-affinity molybdate transporter from *Lotus japonicus*, is essential for molybdate uptake, but not for the delivery to nodules. *Plant J.* 90 1108–1119. 10.1111/tpj.13532 28276145

[B6] Fitzpatrick K. L. Tyerman S. D. Kaiser B. N. (2008). Molybdate transport through the plant sulfate transporter SHST1. *FEBS Lett.* 582 1508–1513. 10.1016/j.febslet.2008.03.045 18396170

[B7] Gao J. S. Wu F. F. Shen Z. L. Meng Y. Cai Y. P. Lin Y. (2016). A putative molybdate transporter LjMOT1 is required for molybdenum transport in Lotus japonicus. *Physiol. Plant* 158 331–340. 10.1111/ppl.12489 27535112

[B8] Gasber A. Klaumann S. Trentmann O. Trampczynska A. Clemens S. Schneider S. (2011). Identification of an Arabidopsis solute carrier critical for intracellular transport and inter-organ allocation of molybdate. *Plant Biol. (Stuttg)* 13 710–718. 10.1111/j.1438-8677.2011.00448.x 21815974

[B9] Gil-Diez P. Tejada-Jimenez M. Leon-Mediavilla J. Wen J. Mysore K. S. Imperial J. (2019). MtMOT1.2 is responsible for molybdate supply to *Medicago truncatula* nodules. *Plant Cell. Environ.* 42 310–320. 10.1111/pce.13388 29940074

[B10] Hibara K.-I. Hosoki W. Hakoyama T. Ohmori Y. Fujiwara T. Itoh J.-I. (2013). *ABNORMAL SHOOT IN YOUTH*, a homolog of molybdate transporter gene, regulates early shoot development in rice. *Am. J. Plant Sci.* 4 1–9. 10.4236/ajps.2013.45a001

[B11] Huang X. Y. Deng F. Yamaji N. Pinson S. R. Fujii-Kashino M. Danku J. (2016). A heavy metal P-type ATPase OsHMA4 prevents copper accumulation in rice grain. *Nat. Commun.* 7:12138. 10.1038/ncomms12138 27387148 PMC4941113

[B12] Huang X. Y. Hu D. W. Zhao F. J. (2021). Molybdenum: more than an essential element. *J. Exp. Bot.* erab534. 10.1093/jxb/erab534 34864981

[B13] Huang X. Y. Liu H. Zhu Y. F. Pinson S. R. M. Lin H. X. Guerinot M. L. (2019). Natural variation in a molybdate transporter controls grain molybdenum concentration in rice. *New Phytol.* 221 1983–1997. 10.1111/nph.15546 30339276

[B14] Ishikawa A. S. Hayashi S. Tanikawa H. Iino M. Abe T. Kuramata M. (2021). Tonoplast-localized OsMOT1;2 participates in interorgan molybdate distribution in rice. *Plant Cell Physiol.* 62 913–921. 10.1093/pcp/pcab050 33826734

[B15] Johnson J. L. Waud W. R. Rajagopalan K. V. Duran M. Beemer F. A. Wadman S. K. (1980). Inborn errors of molybdenum metabolism: combined deficiencies of sulfite oxidase and xanthine dehydrogenase in a patient lacking the molybdenum cofactor. *Proc. Natl. Acad. Sci. U.S.A.* 77 3715–3719. 10.1073/pnas.77.6.3715 6997882 PMC349689

[B16] Jumper J. Evans R. Pritzel A. Green T. Figurnov M. Ronneberger O. (2021). Highly accurate protein structure prediction with AlphaFold. *Nature* 596 583–589. 10.1038/s41586-021-03819-2 34265844 PMC8371605

[B17] Kataoka T. Watanabe-Takahashi A. Hayashi N. Ohnishi M. Mimura T. Buchner P. (2004). Vacuolar sulfate transporters are essential determinants controlling internal distribution of sulfate in *Arabidopsis*. *Plant Cell* 16 2693–2704. 10.1105/tpc.104.023960 15367713 PMC520965

[B18] Kumar S. Asif M. H. Chakrabarty D. Tripathi R. D. Trivedi P. K. (2011). Differential expression and alternative splicing of rice sulphate transporter family members regulate sulphur status during plant growth, development and stress conditions. *Funct. Integr. Genomics* 11 259–273. 10.1007/s10142-010-0207-y 21221698

[B19] Marschner P. Rengel Z. (2012). *Nutrient Availability In Soils,” in Mineral Nutrition of Higher Plants*, 3rd Edn. San Diego, CA: Academic Press, 315–330.

[B20] Schwarz G. (2005). Molybdenum cofactor biosynthesis and deficiency. *Cell. Mol. Life Sci.* 62 2792–2810. 10.1007/s00018-005-5269-y 16261263 PMC11145942

[B21] Schwarz G. Mendel R. R. (2006). Molybdenum cofactor biosynthesis and molybdenum enzymes. *Annu. Rev. Plant Biol.* 57 623–647. 10.1146/annurev.arplant.57.032905.105437 16669776

[B22] Schwarz G. Mendel R. R. Ribbe M. W. (2009). Molybdenum cofactors, enzymes and pathways. *Nature* 460 839–847. 10.1038/nature08302 19675644

[B23] Shibagaki N. Rose A. Mcdermott J. P. Fujiwara T. Hayashi H. Yoneyama T. (2002). Selenate-resistant mutants of *Arabidopsis thaliana* identify *Sultr1;2*, a sulfate transporter required for efficient transport of sulfate into roots. *Plant J.* 29 475–486. 10.1046/j.0960-7412.2001.01232.x 11846880

[B24] Tejada-Jimenez M. Chamizo-Ampudia A. Galvan A. Fernandez E. Llamas A. (2013). Molybdenum metabolism in plants. *Metallomics* 5 1191–1203. 10.1039/c3mt00078h 23800757

[B25] Tejada-Jimenez M. Galvan A. Fernandez E. (2011). Algae and humans share a molybdate transporter. *Proc. Natl. Acad. Sci. U.S.A.* 108 6420–6425. 10.1073/pnas.1100700108 21464289 PMC3080982

[B26] Tejada-Jimenez M. Gil-Diez P. Leon-Mediavilla J. Wen J. Mysore K. S. Imperial J. (2017). *Medicago truncatula* Molybdate Transporter type 1 (MtMOT1.3) is a plasma membrane molybdenum transporter required for nitrogenase activity in root nodules under molybdenum deficiency. *New Phytol.* 216 1223–1235. 10.1111/nph.14739 28805962

[B27] Tejada-Jimenez M. Llamas A. Sanz-Luque E. Galvan A. Fernandez E. (2007). A high-affinity molybdate transporter in eukaryotes. *Proc. Natl. Acad. Sci. U.S.A.* 104 20126–20130. 10.1073/pnas.0704646104 18077439 PMC2148433

[B28] Tomatsu H. Takano J. Takahashi H. Watanabe-Takahashi A. Shibagaki N. Fujiwara T. (2007). An *Arabidopsis thaliana* high-affinity molybdate transporter required for efficient uptake of molybdate from soil. *Proc. Natl. Acad. Sci. U.S.A.* 104 18807–18812. 10.1073/pnas.0706373104 18003916 PMC2141858

[B29] Trumbo P. Yates A. A. Schlicker S. Poos M. (2001). Dietary reference intakes: vitamin A, vitamin K, arsenic, boron, chromium, copper, iodine, iron, manganese, molybdenum, nickel, silicon, vanadium, and zinc. *J. Am. Diet Assoc.* 101 294–301. 11269606 10.1016/S0002-8223(01)00078-5

[B30] Varadi M. Anyango S. Deshpande M. Nair S. Natassia C. Yordanova G. (2021). AlphaFold protein structure database: massively expanding the structural coverage of protein-sequence space with high-accuracy models. *Nucleic Acids Res*. 50 D439–D444. 10.1093/nar/gkab1061 34791371 PMC8728224

[B31] Von Uexküll H. R. Mutert E. (1995). Global extent, development and economic impact of acid soils. *Plant Soil* 171 1–15.

[B32] Wang C. Tang Z. Zhuang J. Y. Tang Z. Huang X. Y. Zhao F. J. (2020). Genetic mapping of ionomic quantitative trait loci in rice grain and straw reveals *OsMOT1;1* as the putative causal gene for a molybdenum QTL *qMo8*. *Mol. Genet. Genomics* 295 391–407. 10.1007/s00438-019-01632-1 31797032

[B33] Xie X. Ma X. Zhu Q. Zeng D. Li G. Liu Y. G. (2017). CRISPR-GE: a convenient software toolkit for CRISPR-based genome editing. *Mol Plant* 10 1246–1249. 10.1016/j.molp.2017.06.004 28624544

[B34] Yan H. Xu W. Xie J. Gao Y. Wu L. Sun L. (2019). Variation of a major facilitator superfamily gene contributes to differential cadmium accumulation between rice subspecies. *Nat. Commun.* 10:2562. 10.1038/s41467-019-10544-y PMC6561962 31189898

[B35] Yang M. Lu K. Zhao F. J. Xie W. Ramakrishna P. Wang G. (2018). Genome-wide association studies reveal the genetic basis of ionomic variation in rice. *Plant Cell* 30 2720–2740. 10.1105/tpc.18.00375 30373760 PMC6305983

[B36] Yoshimoto N. Takahashi H. Smith F. W. Yamaya T. Saito K. (2002). Two distinct high-affinity sulfate transporters with different inducibilities mediate uptake of sulfate in *Arabidopsis* roots. *Plant J.* 29 465–473. 10.1046/j.0960-7412.2001.01231.x 11846879

