# Supplementary material for: The Vacuolar Molybdate Transporter OsMOT1;2 Controls Molybdenum Remobilization in Rice
Source: Front Plant Sci. 2022 Mar 9;13:863816. doi: 10.3389/fpls.2022.863816 (PMC8959823; doi:10.3389/fpls.2022.863816)
Supplement: Supplementary file 1 [file Data_Sheet_1.PDF]

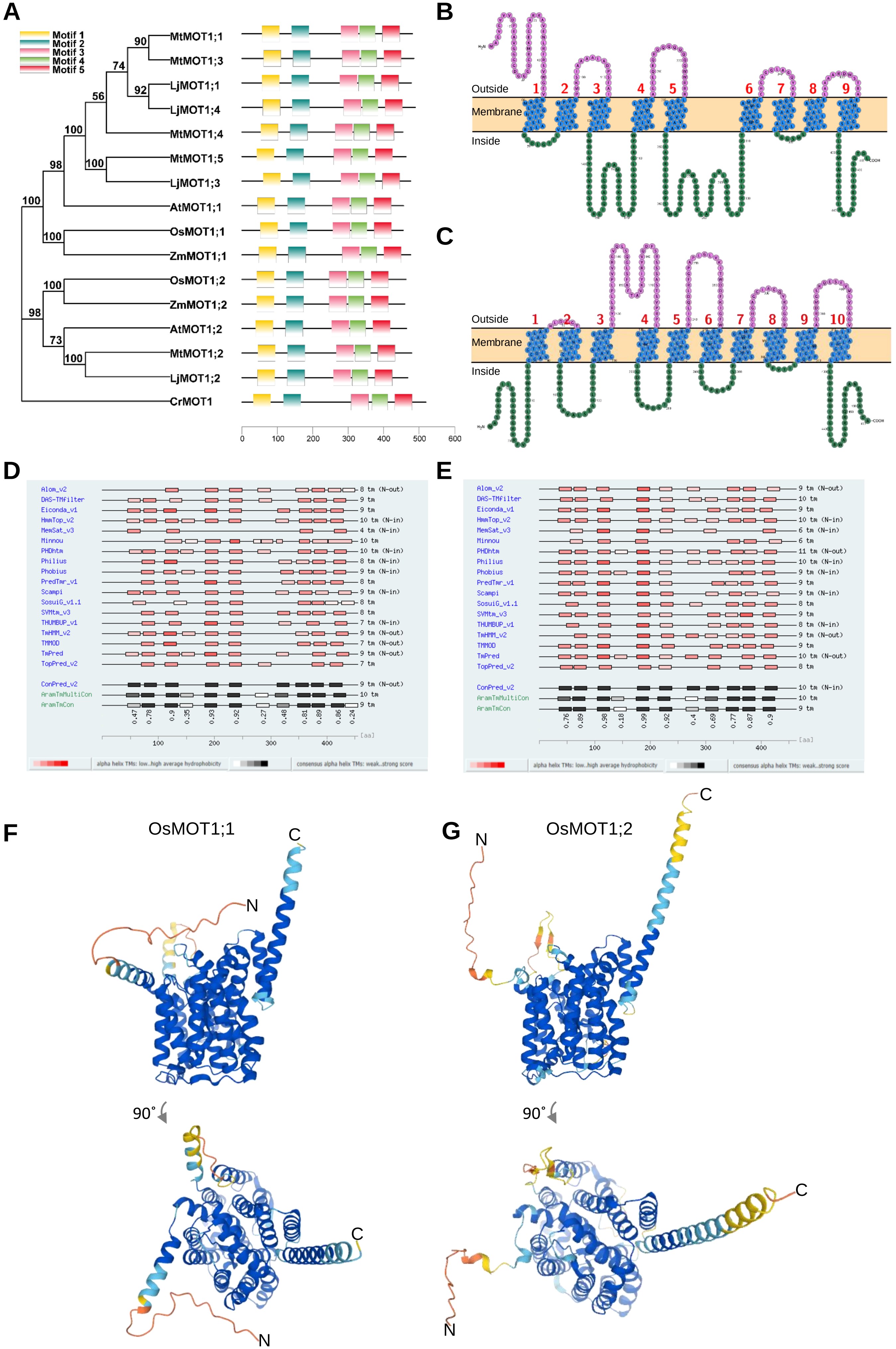

A
Motif 1
Motif 2
Motif 3
Motif 4
Motif 5
MtMOT1;1
MtMOT1;3
LjMOT1;1
LjMOT1;4
MtMOT1;4
MtMOT1;5
LjMOT1;3
AtMOT1;1
OsMOT1;1
ZmMOT1;1
OsMOT1;2
ZmMOT1;2
AtMOT1;2
MtMOT1;2
LjMOT1;2
CrMOT1
90
74
92
56
100
100
98
100
100
100
98
73
100
0
100
200
300
400
500
600
B
Outside
Membrane
Inside
1
2
3
4
5
6
7
8
9
C
Outside
Membrane
Inside
1
2
3
4
5
6
7
8
9
10
D
Alom_v2
DAS-TMfilter
Eiconda_v1
HmmTop_v2
MemSat_v3
Minnou
PHDhtm
Philius
Phobius
PredTmr_v1
Scampi
SosuiG_v1.1
SVMtm_v3
THUMBUP_v1
TmHMM_v2
TMMOD
TmPred
TopPred_v2
ConPred_v2
AramTmMultiCon
AramTmCon
8 tm (N-out)
9 tm
9 tm
10 tm (N-in)
4 tm (N-in)
10 tm
10 tm (N-in)
8 tm (N-in)
9 tm (N-in)
8 tm
9 tm (N-in)
8 tm
8 tm
7 tm (N-in)
9 tm (N-out)
7 tm (N-out)
9 tm (N-out)
7 tm
9 tm (N-out)
10 tm
9 tm
0.47
0.78
0.9
0.35
0.93
0.92
0.27
0.48
0.81
0.89
0.86
0.24
100
200
300
400
[aa]
alpha helix TMs: low..high average hydrophobicity
consensus alpha helix TMs: weak..strong score
E
Alom_v2
DAS-TMfilter
Eiconda_v1
HmmTop_v2
MemSat_v3
Minnou
PHDhtm
Philius
Phobius
PredTmr_v1
Scampi
SosuiG_v1.1
SVMtm_v3
THUMBUP_v1
TmHMM_v2
TMMOD
TmPred
TopPred_v2
ConPred_v2
AramTmMultiCon
AramTmCon
9 tm (N-out)
10 tm
9 tm
10 tm (N-in)
6 tm (N-in)
6 tm
11 tm (N-out)
10 tm (N-in)
9 tm (N-in)
9 tm
9 tm (N-in)
8 tm
9 tm
8 tm (N-in)
9 tm (N-out)
9 tm
10 tm (N-out)
8 tm
10 tm (N-in)
10 tm
9 tm
0.76
0.89
0.98
0.18
0.99
0.92
0.4
0.69
0.77
0.87
0.9
100
200
300
400
[aa]
alpha helix TMs: low..high average hydrophobicity
consensus alpha helix TMs: weak..strong score
F
OsMOT1;1
C
N
90˚
C
N
G
OsMOT1;2
N
C
90˚
C
N

Figure S1. Phylogenetic analysis of MOT1 protein family members and structure predictions of OsMOT1;1 and OsMOT1;2. (**A**) Phylogenetic analysis of MOT1 family proteins and conserved motifs prediction. Accession numbers: AtMOT1;1, At2G25680; AtMOT1;2, At1G80310; CrMOT1, A6YCJ2.1; LjMOT1;1, AFK43331.1; LjMOT1;2, AJE26312.1; MtMOT1;1, XP_013465770.1; MtMOT1;2, XP_024637159.1; MtMOT1;3, XP_013460259.1; MtMOT1;4, XP_013465776.1; MtMOT1;5, XP_003603486.1; OsMOT1;1, LOC_Os08g01120; OsMOT1;2, LOC_Os01g45830; ZmMOT1;1, XP_008664989.1; ZmMOT1;2, NP_001150854.1. (**B**, **C**) Secondary structures of OsMOT1;1 (**B**) and OsMOT1;2 (**C**). The extracellular amino acids are shown in magenta; the transmembrane amino acids are shown in blue; and the cytoplasmic amino acids are shown in green.

(**D**, **E**) Transmembrane domain prediction of OsMOT1;1 and OsMOT1;2. The predictions were performed using the ARAMEMNON 8.1 (http://aramemnon.botanik.uni-koeln.de/index.ep). Up to 18 individual programs were used to predict the transmembrane alpha helices of OsMOT1;1 (**D**) or OsMOT1;2 (**E**). The individual predictions of OsMOT1;1 or OsMOT1;2 were combined to a built-in consensus prediction. The consensus diagram only included the transmembrane segments with consensus scores equal to or above 0.42 according to the instructions of ARAMEMNON. The consensus scores of each transmembrane segments of OsMOT1;1 (**D**) or OsMOT1;2 (**E**) were shown in the bottom in (**D**) or (**E**). TM: transmembrane; N-in: cytoplasmic orientation of N-terminus; N-out: non-cytoplastic orientation of N-terminus.

(**F**, **G**) Three-dimensional (3D) structures of OsMOT1;1 (**F**) or OsMOT1;2 (**G**). The 3D structures were retrieved from AlphaFold Protein Structure Database (www.alphafold.ebi.ac.uk) with the accession numbers: OsMOT1;1, Q6Z1Z0 (E); OsMOT1;2, Q9ASA0 (F). Different colors in the structures of indicated the model confidence defined by a per-residue confidence score (pLDDT) between 0 and 100. Dark blue: very high (pLDDT > 90); sky blue: confident (90 > pLDDT > 70); yellow: low (70 > pLDDT > 50); orange: very low (pLDDT < 50). The structures were shown from the side view (top panel) or the top view (bottom panel).

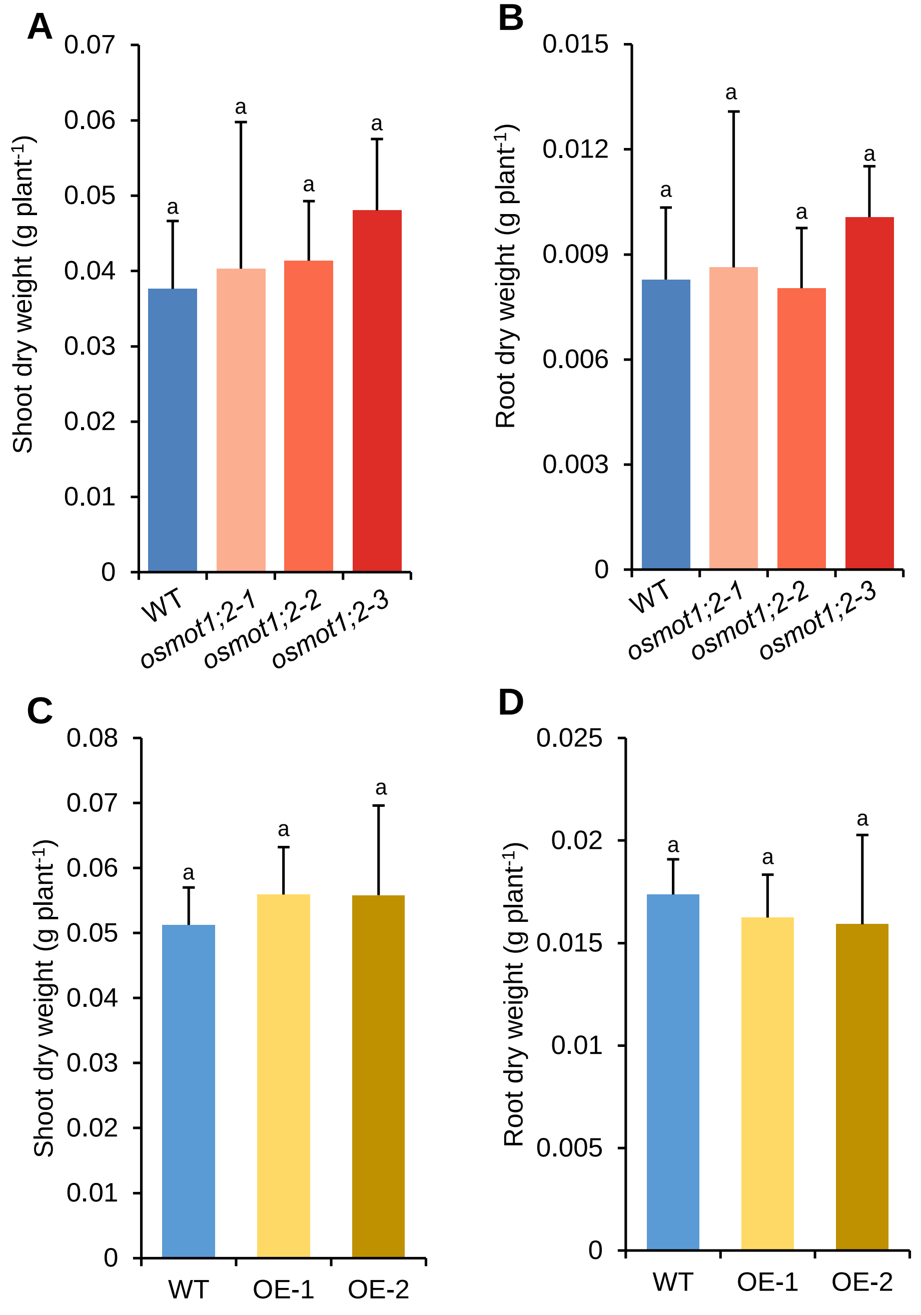

Figure S2. The dry weight of shoots (**A, C**) and roots (**B, D**) of WT, knockout lines (**A**, **B**) and *OsMOT1;2* overexpression lines (**C**, **D**). Plants were cultured with modified half strength Kimura B nutrient solution with 10 nM Mo until four leaves seedling stage. Data are presented as means ± SD with five biological replicates. Columns with different letters in indicate significant differences at $P < 0.05$ (Tukey's honestly significant difference test).

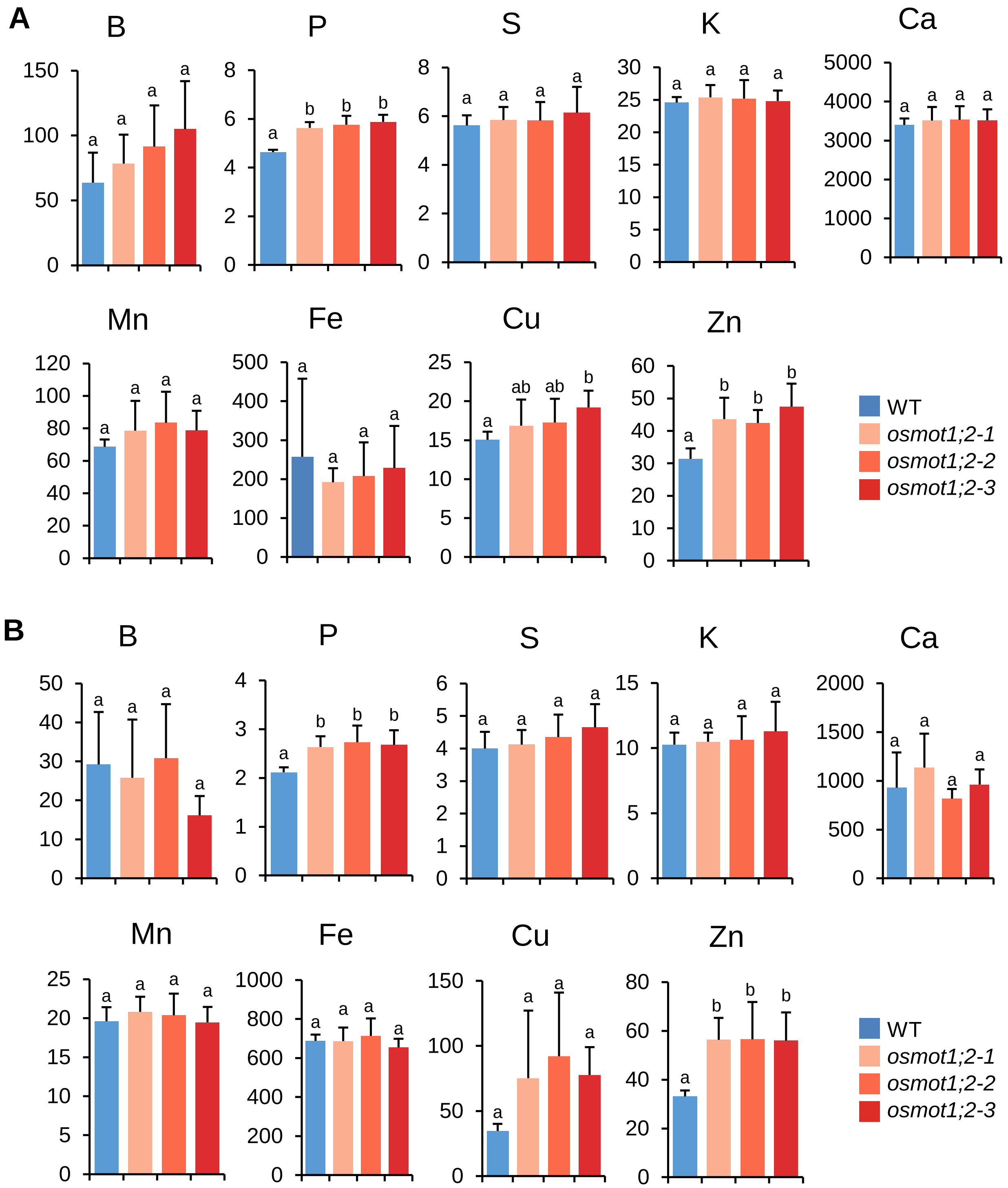

Figure S3. Concentrations of various elements in shoots and roots of WT and *osmot1;2* at four leaves seedling stage. A, shoots; **B**, roots. The unit of B, Ca, Mn, Fe, Cu and Zn concentrations in shoots and roots is $\mu g\ g^{-1}$ DW, and the unit of P, S and K concentrations in shoots and roots is $mg\ g^{-1}$ DW. Data are presented as means ± SD with six biological repeats. Columns with different letters indicate significant differences at $P < 0.05$ (Tukey's honestly significant difference test).

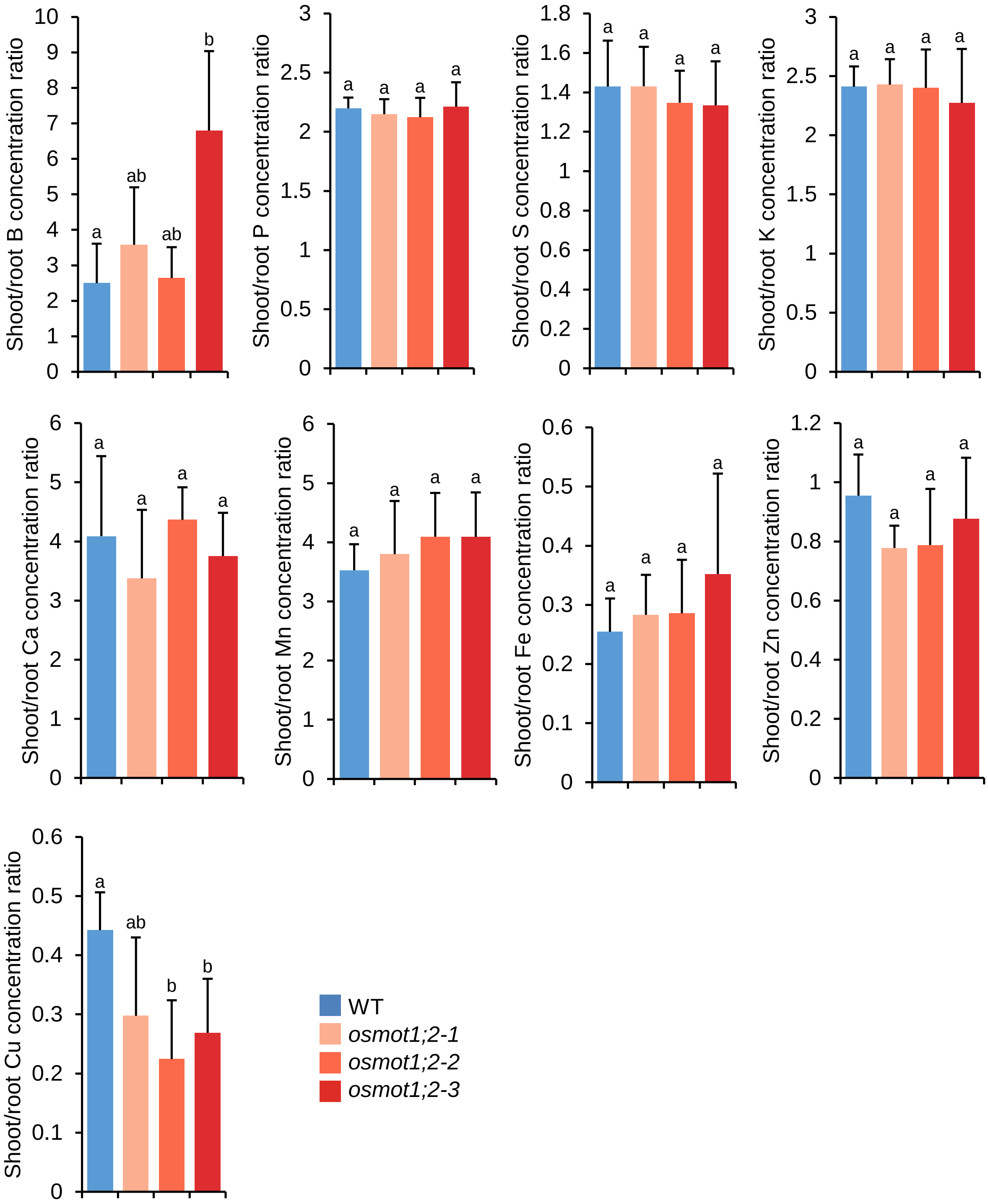

Figure S4. The shoot/root concentration ratio of 9 elements in the WT and *osmot1;2*. Data are presented as means ± SD with five or six biological repeats. Columns with different letters indicate significant differences at $P < 0.05$ (Tukey's honestly significant difference test).

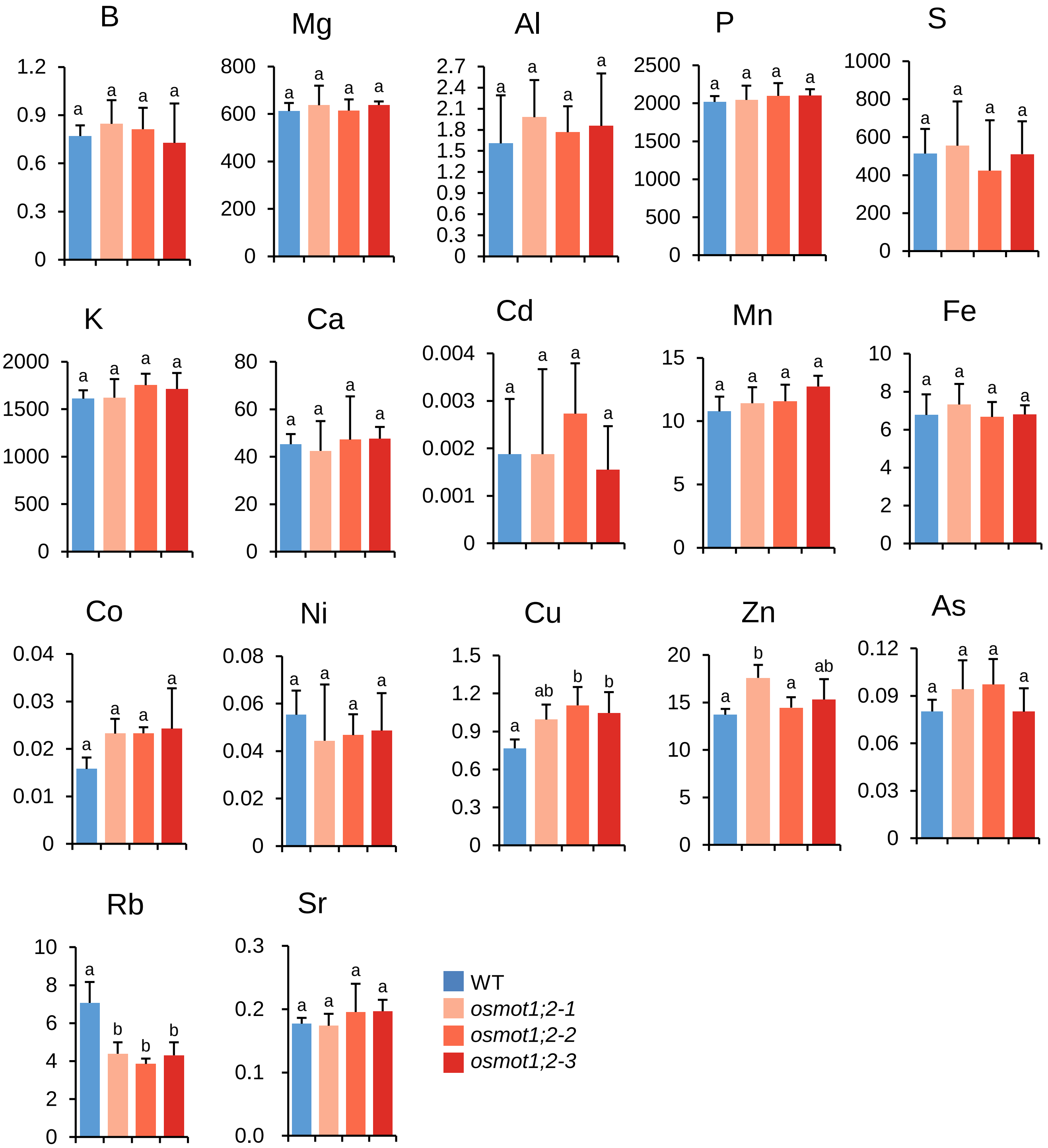

Figure S5. Concentrations of various elements in grains of WT and *osmot1;2*. The unit of all nutrient elements concentration in grains is μg g^{-1} DW. Data are presented as means ± SD with five biological repeats. Columns with different letters indicate significant differences at $P < 0.05$ (Tukey's honestly significant difference test).

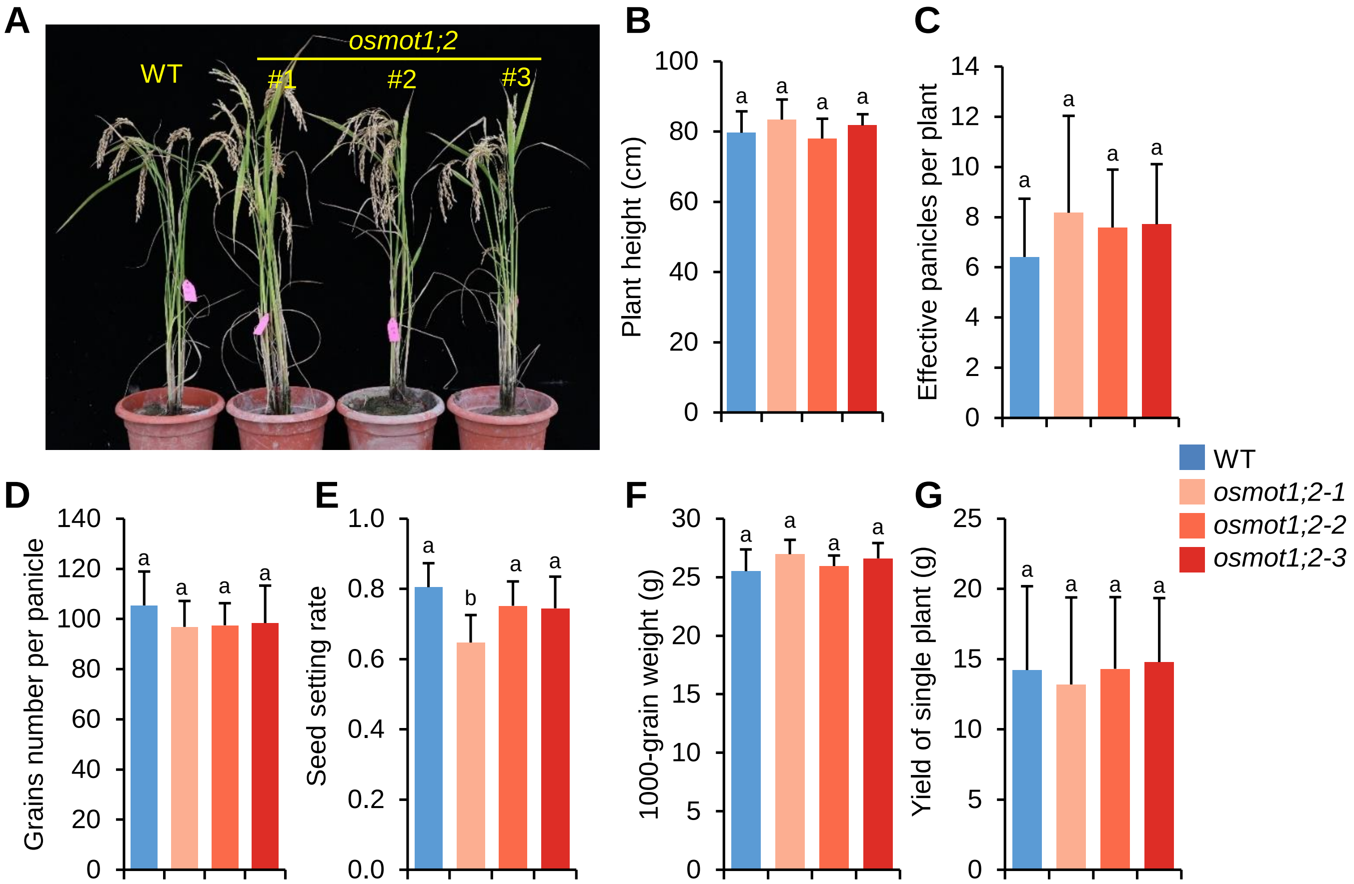

Figure S6. The phenotype and agronomic traits of WT and *osmot1;2*. (A) The growth phenotype of WT and three independent knockout lines of *OsMOT1;2* at harvesting stage. **(B)** Plant height. **(C)** Effective panicles per plant. **(D)** Grain number per panicle. **(E)** Seed setting rate. **(F)** 1000-grain weight. **(G)** Yield per plant. Data are presented as means ± SD with six-fourteen biological repeats. Columns with different letters in (**B**-**G**) indicate significant differences at $P < 0.05$ (Tukey's honestly significant difference test).

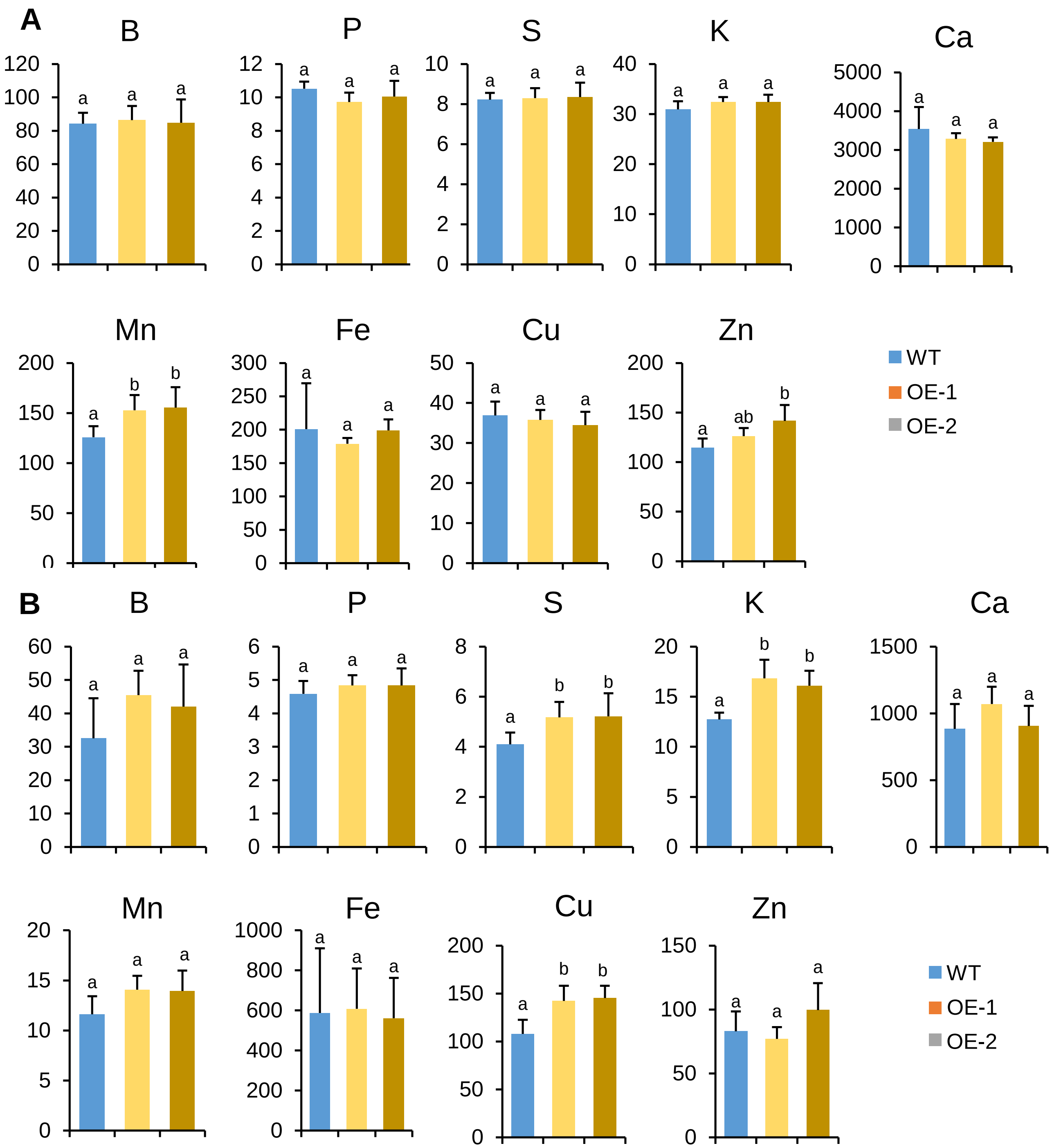

Figure S7. Concentrations of various elements in shoots and roots of WT and overexpression lines of *OsMOT1;2* at four leaves seedling stage. A, shoots; **B**, roots. The unit of B, Ca, Mn, Fe, Cu and Zn concentration in shoots and roots is $\mu g\ g^{-1}$ DW, and the unit of P, S and K concentration in shoots and roots is $mg\ g^{-1}$ DW. Data are presented as means ± SD with six biological repeats. Columns with different letters indicate significant differences at $P < 0.05$ (Tukey's honestly significant difference test).

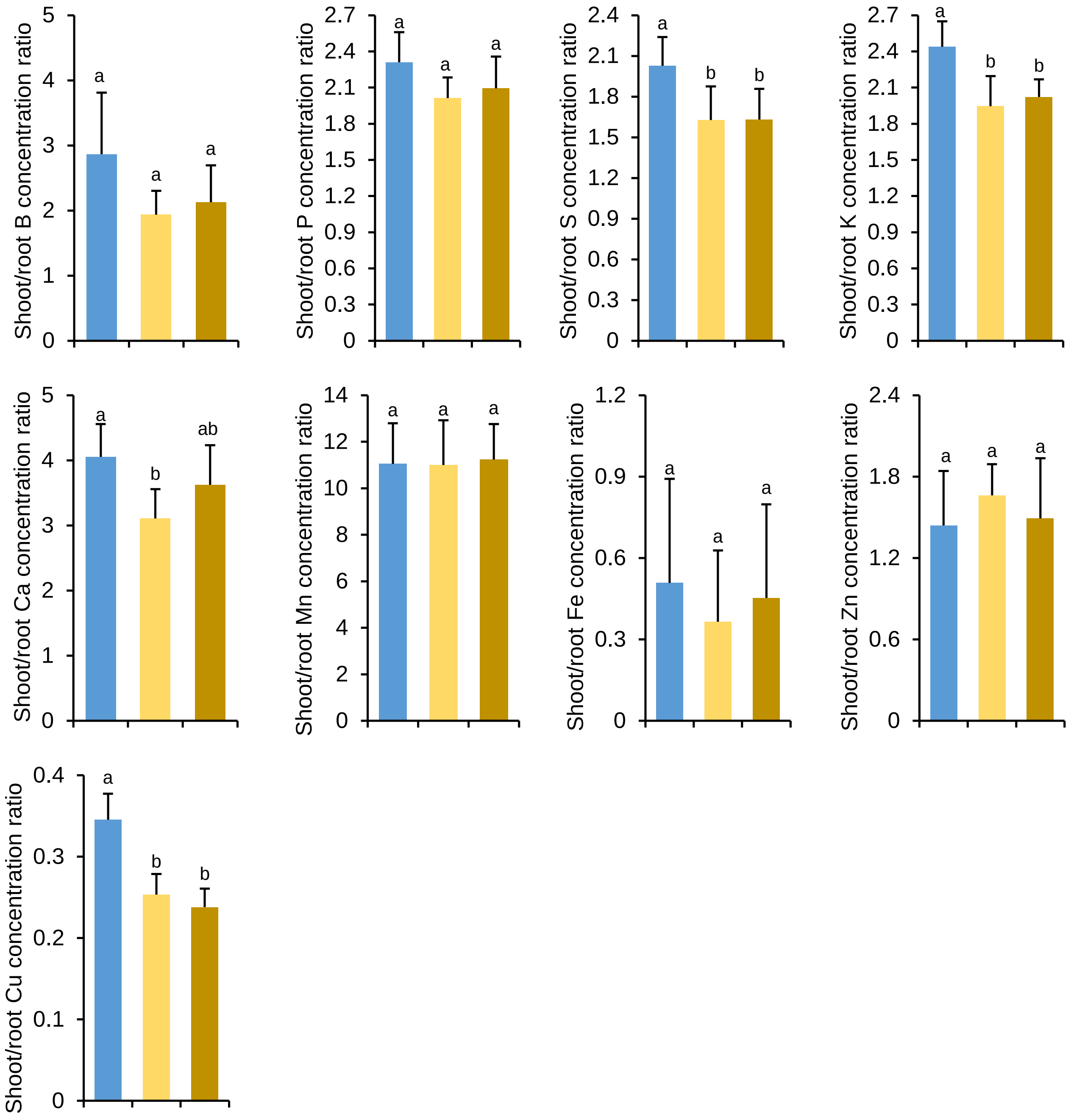

Figure S8. The shoot/root concentration ratio of 9 elements in the WT and *OsMOT1;2*-overexpression lines. Data are presented as means ± SD with six biological repeats. Columns with different letters indicate significant differences at $P < 0.05$ (Tukey's honestly significant difference test).

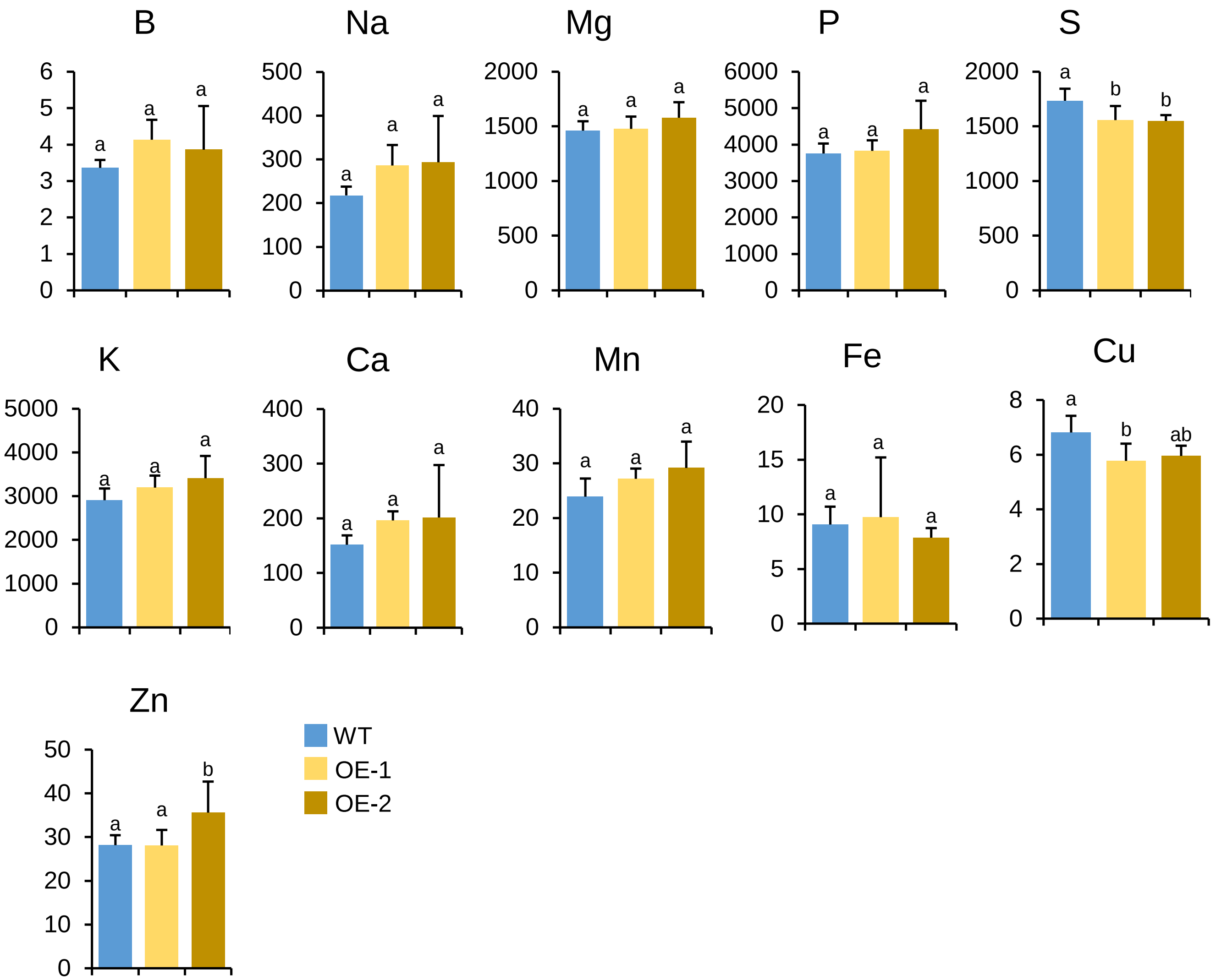

Figure S9. Concentrations of various elements in grains of WT and *OsMOT1;2* overexpression lines. The unit of all nutrient elements concentration in grains is μg g^{-1} DW. Data are presented as means ± SD with six biological repeats. Columns with different letters indicate significant differences at $P < 0.05$ (Tukey's honestly significant difference test).

Table S1. The primers used in this study.

Purpose of the experiment	Primer name	Sequence (5'-3')
Construction of CRISPR/Cas9 knockout vector	Grt1	TAGCGGATGTACTTGACGGGTTTTAGAGCTAGAAAT
	OsU6at1	CCGTCAAGTACATCCGCTACGGCAGCCAAGCCAGCA
	Grt2	AGGATTATGAACAGTAGCGGTTTTAGAGCTAGAAAT
	OsU6bt2	CGCTACTGTTCATAATCCTCAACACAAGCGGCAGC
Generation of *OsMOT1;2* overexpression lines	pUN-eGFP-Os1;2-F1	CGACTCTAGAGGATCCATGGTGAGCAAGGGCGAG
	pUN-eGFP-Os1;2-R1	GATGCCATCTTGTACAGCTCGTCCATGCC
	pUN-eGFP-Os1;2-F2	GTACAAGATGGCATCCTCCGCCG
	pUN-eGFP-Os1;2-R2	GATCGGGGAAATTCGAGCTCTCAAGCATCTCCAGCCCCATC
Detect mutations of *OsMOT1;2* sequence	CRISPROs1;2-F	CCCCATGCCCGTCCAGCCCAT
	CRISPROs1;2-R	CGGCGCCCTCCCAGAACCCGATCT
qRT-PCR	Act-rts	TGGTCGTACCACAGGTATTGTGTT
	Act-rta	AAGGTCGAGACGAAGGATAGCAT
	qOsMOT1;2-F	CTCATGAATTTCGTGGGGTG
	qOsMOT1;2-R	AGCATGACGCCCAGTATC
Promoter GUS construct	GUS-Os1;2-F	CATGCGGCCGCTTAATTAAGTTATACCATCTGGAGTTACGCCATGC
	GUS-Os1;2-R	CCTTTGCACGGCGCGCCGGGTGGGAAACTCGAAAGCAACGACT
Heterologous expression in yeast	pDROsMOT1;2-F	CGGGCTGCAGGAATTCATGGCATCCTCCGCCG
	pDROsMOT1;2-R	CGGGCCCCCCCTCGAGTCAAGCATCTCCAGCCCCATCT
	pDRAtMOT1;2-F	CGGGCTGCAGGAATTCATGGAGACAACTACAACTCCTCTGC
	pDRAtMOT1;2-R	CGGGCCCCCCCTCGAGTTAGACATCACGAGGAGCGGCT
Subcellular localization	A7GFP-Os1;2-F	TGTACAAGGGTCTAGACATGGCATCCTCCGCCG
	A7GFP-Os1;2-R	CGATCAATCAGGATCCTCAAGCATCTCCAGCCCCATC
